# Ocean Transport Pathways to a World Heritage Fringing Coral Reef: Ningaloo Reef, Western Australia

**DOI:** 10.1371/journal.pone.0145822

**Published:** 2016-01-20

**Authors:** Jiangtao Xu, Ryan J. Lowe, Gregory N. Ivey, Nicole L. Jones, Zhenlin Zhang

**Affiliations:** 1 School of Civil, Environmental and Mining Engineering, The University of Western Australia, Crawley, Australia; 2 The UWA Oceans Institute, The University of Western Australia, Crawley, Australia; 3 School of Earth and Environment, The University of Western Australia, Crawley, Australia; 4 ARC Centre of Excellence for Coral Reef Studies, The University of Western Australia, Crawley, Australia; University of Lincoln, UNITED KINGDOM

## Abstract

A Lagrangian particle tracking model driven by a regional ocean circulation model was used to investigate the seasonally varying connectivity patterns within the shelf circulation surrounding the 300 km long Ningaloo Reef in Western Australia (WA) during 2009–2010. Forward-in-time simulations revealed that surface water was transported equatorward and offshore in summer due to the upwelling-favorable winds. In winter, however, water was transported polewards down the WA coast due to the seasonally strong Leeuwin Current. Using backward-in-time simulations, the subsurface transport pathways revealed two main source regions of shelf water reaching Ningaloo Reef: (1) a year-round source to the northeast in the upper 100 m of water column; and (2) during the summer, an additional source offshore and to the west of Ningaloo in depths between ~30 and ~150 m. Transient wind-driven coastal upwelling, onshore geostrophic transport and stirring by offshore eddies were identified as the important mechanisms influencing the source water origins. The identification of these highly time-dependent transport pathways and source water locations is an essential step towards quantifying how key material (e.g., nutrients, larvae, contaminants, etc.) is exchanged between Ningaloo Reef and the surrounding shelf ocean, and how this is mechanistically coupled to the complex ocean dynamics in this region.

## Introduction

**Fig 1:**
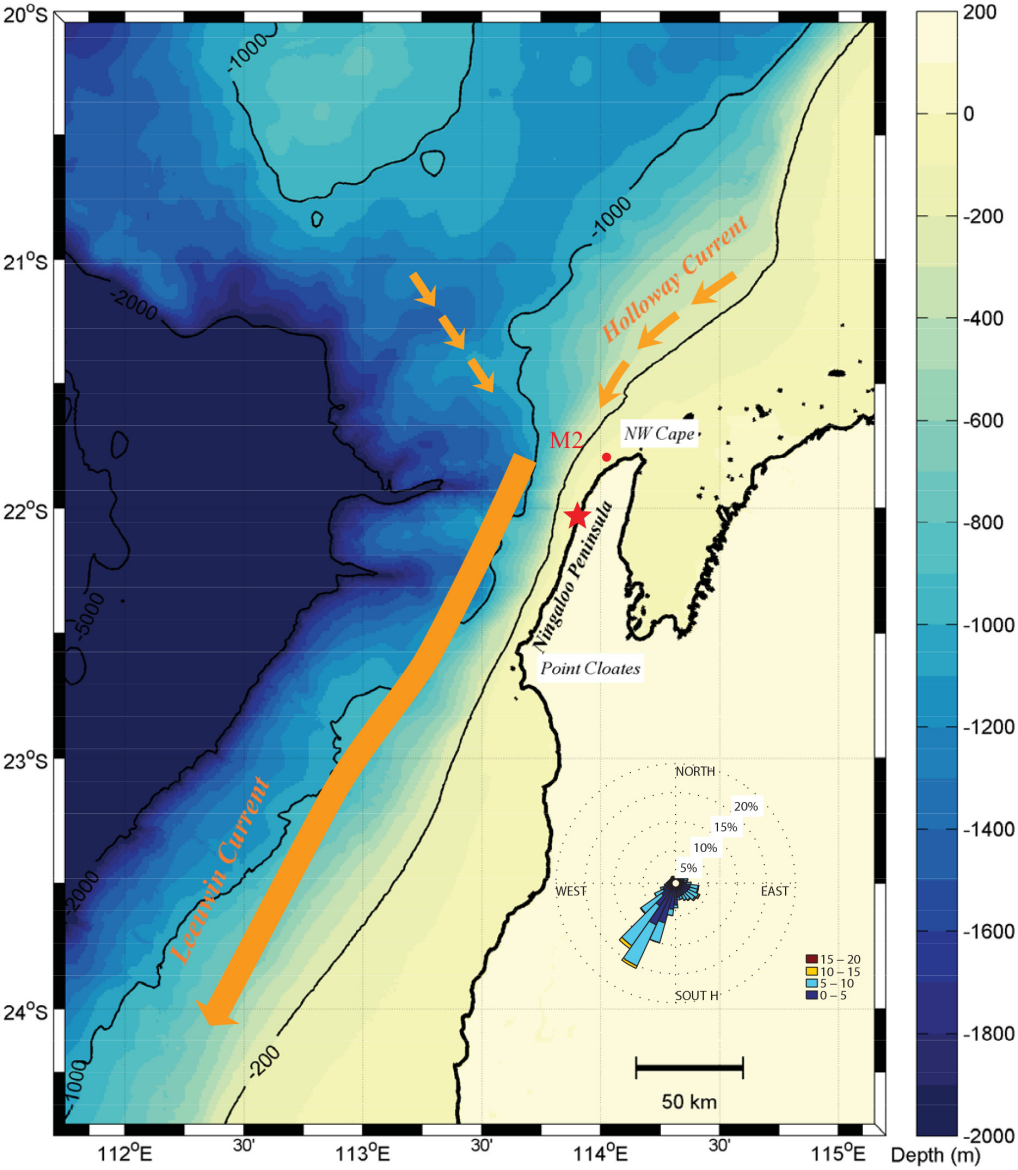
Map of the study area, showing both the bathymetry contours (colorbar in meters) and a schematic of the main upper ocean current systems that can influence the Ningaloo shelf. The red dot denotes the M2 mooring off the Ningaloo Peninsula. The wind rose shows the statistical distribution of the wind direction (oriented in the direction the winds come from) and wind magnitude (colored bars in m s^-1^), calculated using two years (2009–2010) of wind data collected at a weather station maintained by the Australian Institute of Marine Science (located at the red star).

Ningaloo Reef in Western Australia (WA) is the world’s largest fringing coral reef system and a United Nations World Heritage site that supports a wide range of habitats and a high diversity of marine organisms (Fig 1). Like other coral reefs, the ocean circulation surrounding Ningaloo Reef regulates how material (e.g., nutrients, larvae, etc.) and heat are exchanged between the reef and ocean, which in turn shapes the ecology of this reef system. Understanding the detailed transport pathways, including identifying the primary source and sink regions of the coastal water that exchanges with Ningaloo Reef, can thus help address many critical questions, such as: How connected is Ningaloo Reef to other reef systems along the west coast of Australia?; How sensitive is this reef ecosystem to climate-driven variability of the ocean circulation and associated heat transport?; and, How vulnerable is Ningaloo to increasing human pressures, including those from one of the world’s most rapidly growing offshore resource industry directly to its north?

The ocean circulation off Ningaloo Reef is shaped by the unique large-scale ocean dynamics that also drive the shelf circulation along the broader coastline of WA [1]. In particular, the region experiences an unusually strong poleward-directed meridional pressure gradient that opposes the dominant equatorward wind stresses (Fig 1), which is a unique phenomenon among the world’s major eastern boundary current systems [2–4]. Historically, the shelf dynamics along WA have been most well-studied in the southwest of Australia [4–8], located several hundred kilometers to the south of Ningaloo Reef. These studies have shown how the pressure gradient-driven Leeuwin Current (hereafter referred as the LC) transports warm and low salinity water from the tropics down the WA coast [2, 6]. Based on a large-scale Langrangian particle tracking study of the entire eastern Indian Ocean using a global-scale ocean model, Domingues et al. [8] provided insight into the large-scale source water origins that contribute to the formation of the LC. They argued that the northern source of the LC is largely from the tropical equatorial Indian Ocean via the south Java Current, with an additional component from the Pacific Ocean water via the Indonesian Throughflow. Measurements during May-June 1993 reported by Holloway [7] indicated that there may also be a significant south-westward flow on the Australian North West Shelf (hereafter referred as NWS) towards Ningaloo Reef, which is now commonly referred to as the ‘Holloway Current’. Recent observations specifically off the Ningaloo Peninsula (the region between the North West Cape and Point Cloates; Fig 1) in the austral spring [9] have suggested that part of LC water originates from a local eastward-directed onshore geostrophic flow, which in turn forms a poleward along-shelf flow down the coast of the Ningaloo Peninsula.

The wind patterns in the WA region are predominantly upwelling-favourable (towards the northeast, see Fig 1), although they also display some seasonality: winds are on average strongly north-eastward during the austral summer, weaker but still towards the northeast in spring and autumn, and weak and more variable in direction during winter [1, 8]. When local wind stresses are strong enough to overcome the persistent along-shelf pressure gradients in the region, transient coastal upwelling over the full length of the Ningaloo Peninsula often occurs [9]. During summer months, coastal upwelling has been shown to substantially reduce water temperatures along Ningaloo Reef by on average 2–3°C relative to offshore during summer [1], which may explain why Ningaloo has experienced only very limited thermal coral bleaching relative to other reefs in the eastern Indian Ocean. Over most of the year, the dominant equatorward winds also tend to interact with the opposing poleward pressure gradient, creating highly energetic mesoscale eddy fields that can encroach towards the coast [10, 11]. Both transient coastal upwelling and eddies are thought to provide substantial fluxes of deep-water nutrients to Ningaloo Reef [12, 13] and also stimulate local phytoplankton blooms along the Ningaloo shelf; for example, frequent observations of a band of high chlorophyll-a concentration (a proxy for phytoplankton biomass) adjacent to the Ningaloo shelf have been attributed to localized upwelling [12]. Vertical fluxes of nutrient have also been found to be enhanced within (sub)mesoscale eddies during opportunistic ship-based transects off Ningaloo [14]. However, due to the complex hydrodynamic processes that occur in the Ningaloo region, the mechanisms by which water masses (e.g. carrying nutrients) interact with this reef system still remain poorly quantified, yet are believed to be key to understanding why this fringing reef system is so productive along the generally oligotrophic coast of WA [12, 13].

Langrangian particle tracking provides a useful approach to assess the connectivity pathways both to and from different oceanic regions [15, 16]. Using particle tracking driven by a regional ocean circulation model, this study focuses on identifying the key source regions of water that is advected both to and from Ningaloo Reef during different seasonal periods (specifically focusing on a period during 2009–10), including how various hydrodynamic mechanisms contribute to the observed transport. The paper is organized as follows. We first describe the methodologies in Section 2, including the setup of the hydrodynamic model and the Lagrangian particle tracking model. In Section 3 we describe the seasonal shelf circulation, and in Section 4 use the particle tracking results to assess the three-dimensional transport pathways during four different seasons. In Section 5, the mechanisms responsible for these transport pathways are discussed as well as the broader implications of the results (e.g. identifying potential nutrient source regions). Finally, we summarize the main results in Section 6.

## Methodology

### 2.1 Circulation model

**Fig 2:**
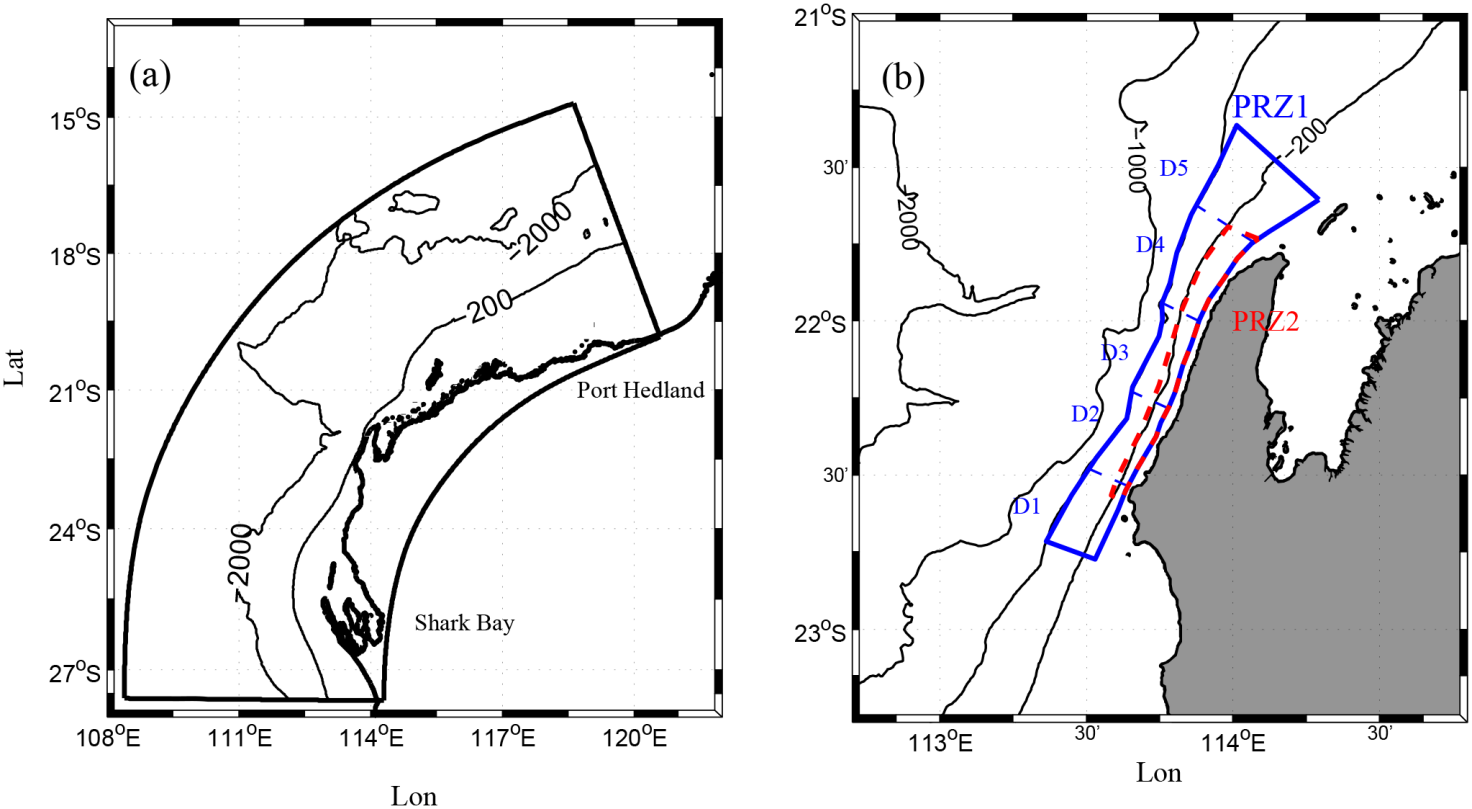
Model domain along the NWS surrounding Ningaloo Reef. (a) the ROMS hydrodynamic model domain; (b) the particle release zones in Scenario 1, Scenario 2 (blue box, denoted as region PRZ1), as well as in Scenario 3 (red box, denoted as region PRZ2) on the Ningaloo shelf. The five sub-domains within PRZ1 in (b) are termed D1, D2, D3, D4 and D5, from south to north, separated by blue dashed lines.

The Regional Ocean Modeling System (ROMS) is a terrain-following, hydrostatic primitive equation ocean model that is widely used by the ocean modelling community [17]. ROMS was used to simulate the circulation of the NWS region of Australia surrounding Ningaloo Reef, using a similar model set up to that detailed in Xu et al. [18]. The model was configured using a curvilinear grid, extending roughly 1000 km along the coast and 500 km offshore (Fig 2). The variable horizontal-resolution grid was relatively coarse in offshore regions (~4 km) and finer near the coast (down to ~1 km). Vertically, the water column was discretized into 40 sigma layers, with higher resolution near the surface and bottom (e.g. at the 100 m isobath, the surface layers were ~0.1 m thick and the bottom layers were ~5 m thick).

To investigate the seasonal variability in the transport paths, we conducted simulations for four periods covering a year period from Nov 2009 to Nov 2010: (1) 1 Nov 2009 to 1 Jan 2010, hereafter referred to as “summer”; (2) 1 Mar 2010 to 1 May 2010, hereafter referred to as “autumn”; (3) 1 May 2010 to 1 Jul 2010, hereafter referred to as “winter”; and (4) 1 Sep 2010 to 1 Nov 2010, hereafter referred to as “spring”. These periods were chosen since they cover the detailed field mooring data recorded on the Ningaloo shelf as described in Xu et al. [9]. Previous work by Xu et al. [18] showed that, in the absence of data assimilation, model simulations over this duration ensured negligible long-term model drift from their initial conditions. Separate ROMS runs were thus conducted for four separate seasonal simulations, and the resulting three-dimensional velocity fields were used to drive the particle tracking models (described below) for each period.

Although the model was configured similarly to that described in Xu et al. [18], for the present study we included the surface heat fluxes using atmospheric data obtained from the Climate Forecast System Reanalysis (CFSR) results archived at the National Operational Model Archive and Distribution System (NOMADS, http://nomads.ncdc.noaa.gov/) with 0.3° resolution. We initialized and drove the model at the lateral open boundaries using global hindcast reanalysis output from a hybrid coordinate ocean model (HYCOM, 1/12°, www.hycom.org/ [19]). This approach allows the large-scale and remotely-generated dynamics (including currents, temperature/salinity fields, and sea surface height) to drive the regional ROMS model. Nonetheless, we previously found HYCOM tended to over-predict the strength of the poleward flow during the Nov-Dec 2009 period [18]. Thus, we instead used direct satellite sea surface height observations from Australia’s Integrated Marine Observing System (IMOS, http://thredds.aodn.org.au/thredds/catalog/IMOS/OceanCurrent/GSLA/catalog.html) to initialize the surface elevation fields in the ROMS model. The three-dimensional velocity outputs were, in turn, used to drive the particle tracking models (described below) for each period.

### 2.2 Particle tracking model

To simulate the time-integrated transport pathways using particle tracking, we used the offline Larval Transport Lagrangian model (LTRANS [20], see http://northweb.hpl.umces.edu/LTRANS.htm). Although LTRANS was initially developed to simulate active and passive larval transport [21], it has since been successfully applied to a number of other applications; for example, to assess oil droplet dispersal from the Deep Water Horizon oil-spill in the Gulf of Mexico [22]. LTRANS reads and interpolates ROMS three-dimensional velocity vector fields onto particle positions at each hydrodynamic model output time step (external time step). Based on these interpolated values, the model calculates particle movements and updates their positions in three-dimensional space at each internal time step using a passive particle displacement algorithm that includes transport from both advection and turbulent diffusion, as:

$$x_{n+1}=x_{n}+u\delta t+R{\left[2r^{-1}K_{h}\delta t\right]}^{1/2}$$ (1)

$$y_{n+1}=y_{n}+v\delta t+R{\left[2r^{-1}K_{h}\delta t\right]}^{1/2}$$ (2)

$$z_{n+1}=z_{n}+w\delta t+\frac{\partial K_{v}}{\partial z}\delta t+R{\left[2r^{-1}K_{v}\delta t\right]}^{1/2}$$ (3)

where *K*_*v*_ and *K*_*h*_ are the vertical and horizontal diffusivities derived from the ROMS model, respectively; *δt* is the internal time step for the Lagrangian particle tracking; *R* is a random number generator with mean = 0 and standard deviation *r* = 1; *x*_*n*_ and *y*_*n*_ denote the horizontal particle positions at the *n*^th^ time step, and *z*_*n*_ the vertical positions at the *n*^th^ time step. The advection of particles was computed by numerically integrating the velocities using a 4^th^ order Runge-Kutta scheme. In our simulations, LTRANS read the ROMS output every 6 h (external time step), and predicted and updated the particle displacements every 3 minutes (internal time step). Like most particle tracking models, the built-in random displacement module [23, 24] based on turbulent diffusivities from ROMS may not fully represent realistic turbulent mixing processes in the coastal ocean. We examined the influence of the random displacement module on particle trajectories separately, as detailed in Appendix A. These results indicate that the dominant transport pathways are largely insensitive to sub-grid scale turbulent diffusion processes, consistent with a much stronger role of advection on the overall transport.

On this basis, by reversing the input sequence in time and the direction of the velocity fields from the ROMS simulations, we ran LTRANS backward-in-time to determine the source of the water parcels reaching Ningaloo Reef. The benefits of backward-in-time tracking (hereafter referred as BITT) [16] become most apparent when the size of sink destinations in a model is significantly smaller than that of the potential sources—as in the present application. Therefore, with a fixed number of particles within the sink destination region, we were able to track and identify the source region of each particle with much greater efficiency compared to applying conventional forward-in-time tracking (hereafter referred as FITT) over all grid cells within the entire domain. Since diffusion processes are irreversible, we switched off the particle turbulence sub-grid scale model in order to track the particle movements due to advection alone. These advection-only FITT and BITT simulations should be reversible, which was confirmed by a set of test runs (see Supporting Information, S1 Fig).

### 2.3 Particle initialization, source tracking, and transport scenarios

Three particle tracking scenarios were considered in this study (see Table 1). Scenario 1 investigated the two-dimensional horizontal surface flow pathways originating from Ningaloo Reef during the four different seasons. In this scenario, particles were released at the surface (0 m depth) and trajectories were calculated over 60-day simulations for each season. The Scenario 1 simulations were run forward as FITT simulations. Scenario 2 investigated the reverse dynamics, i.e., the source locations of water masses that reached the Ningaloo shelf during the different seasons. In these simulations, particles were released at two depths (10 m and 50 m below the surface) adjacent to the coast and were tracked backwards using BITT simulations for 60 days. Finally, in Scenario 3, the effects of shorter-term (order weekly) local wind forcing events on vertical particle excursions were investigated using a series of shorter BITT simulations. Since the wind events responsible for driving transient upwelling or downwelling in the Ningaloo region tend to occur within a synoptic weather band with a period of ~10 days [1, 18, 25, 26], particles were initialized at a depth of 10 m near the coast, and six separate 10-day BITT simulations were conducted within each seasonal period.

**Table 1:** The configurations for the three particle tracking scenarios.

	Model type	Duration	Number of simulations	Initial depth	Initial domain	Turbulence module
Scenario1	FITT	2 months	1 in each season	surface (0 m)	PRZ1	RDM^#^
Scenario2	BITT	2 months	1 in each season	10m	PRZ1	N/A
				50m	PRZ1	N/A
Scenario3	BITT	10 days	6 in each season	10m	PRZ2	N/A

^#^ RDM denotes the random displacement module.

For Scenarios 1 and 2, particles were released within particle release zone 1 (hereafter PRZ1), denoted by the blue polygon in Fig 2, which extended along the entire Ningaloo peninsula with width ~20 km and an outer boundary coinciding with the 600 m isobath. For Scenario 3, we released particles within a narrower zone, with an outer boundary at the 200 m isobath (referred to as PRZ2, ~7 km width), denoted by the red polygon in Fig 2 that coincided with the typical coastal upwelling zone width at the site [18]. The FITT model (in Scenario 1) was run with the random displacement module, but was disabled in the BITT simulations (in Scenarios 2 and 3). For all simulations, once a particle moved outside the model domain it was no longer tracked. Finally, to avoid confusion when reporting the tracking results below, we present the transport paths in the BITT simulations in a forward-in-time perspective, to maintain consistency among all three scenarios. Therefore, particles “originate” from the final model positions and arrive at their “initial” positions at the beginning of the BITT simulations.

### 2.4 Transport analysis

To determine the source origins of water transported to different areas along Ningaloo Reef for Scenario 2, we further divided the particle release zone into five sub-domains enclosed within the 600 m isobath (regions D1–D5), and released ~7000 particles to examine the mean transport pathways to each sub-domain (see Fig 2b). To investigate the vertical transport of particles due to coastal upwelling in Scenario 3, we calculated the average vertical migration of the particles over an inshore PRZ2 domain (Fig 2b) for each 10-day simulation period. These computed vertical displacements were then compared with those estimated from an analytical model of coastal upwelling, where the mean vertical upwelling velocity *w* within an upwelling zone is [27, 28]:

$$w=\overset{Ekman}{\overset{︷}{\frac{-\tau_{xs}}{\rho_{0}fL}}}+\overset{geostrophic}{\overset{︷}{\frac{v_{g}H}{L}}}$$ (4)

Here *ρ*_0_ is the reference seawater density, *τ*_*xs*_ denotes the along-shelf wind stress with positive sign directed equatorward (northeastward) along the Ningaloo peninsula (refer to Xu et al. [18]) and *f* is the Coriolis parameter (negative in the southern hemisphere). In (Eq 4), the first term is the vertical velocity contribution from the along-shelf winds (i.e. coastal upwelling due to surface Ekman transport), which has been found to be a good description of wind-driven cross-shelf transport (after removing the background interior flow) along the Ningaloo shelf [9, 18]. The second term represents a geostrophic correction of the contribution from any background barotropic cross-shelf flow. The real ocean barotopic cross-shelf flow can be spatially-complex and transient, e.g. due to eddies and meandering shelf currents with order daily-weekly temporal variability. These higher-frequency dynamics can only be resolved using three-dimensional ocean circulation models, as in the present study. Alternatively, Marchesiello et al. [28] proposed a simple model incorporating a climatological cross-shelf barotropic geostrophic flow to estimate the impact of this flow on the upwelling/downwelling transport in (Eq 4) in the absence of a numerical model. This method has been previously used to predict variability in upwelling/downwelling along the WA coast (including along Ningaloo Reef) by Rossi et al. [25], and although it has been shown to qualitatively capture a number of major historical upwelling events (i.e., as inferred from satellite imagery), it has not yet been verified with direct in situ observations or numerical model simulations of vertical transport. We thus used this analytical model as a framework to evaluate how different mechanisms contribute to upwelling/downwelling, and moreover, assessed how this simple analytical model (incorporating only climatological information about the background geostrophic flow) performed against our highly-resolved 3D numerical simulations. Therefore, in (Eq 4) we treated *v*_*g*_ as the cross-shelf geostrophic velocity (with positive velocity directed offshore), which was estimated by a downward integration of the pressure gradient above the 100 dbar reference level from the CARS09 temperature and salinity seasonal climatological dataset [25], where *H* is the surface layer depth taken as the mixed layer depth from CARS09. The cross-shelf width of the upwelling zone *L* was taken as the mean distance between the coastline and the 200 m isobath, also corresponding to the mean width of the PRZ2 zone. Although the buoyancy frequency generally varies from ~0.003 s^-1^ in winter to ~0.01 s^-1^ in summer on the Ningaloo shelf [9], and the surface layer depth ranges from ~100 m (winter) to ~20 m (summer), the baroclinic Rossby radius (defined as *NH*/ *f*) is on average ~ 7 km year-round and shows only weak seasonality, hence coinciding with our estimate of *L*. Notably the width of the upwelling zone used in Marchesiello et al. [28] (*L* = 0.75*D*/*S*, where *D* is mixed layer depth and *S* is the shelf slope) is only valid for a shallow shelf where bottom friction limits the development of an upwelling front, while for the steep shelf slope at Ningaloo, the standard definition based on the baroclinic Rossby radius is more applicable. The average vertical displacement *h*' of water parcels during each 10-day analysis period can thus be estimated from the integration of (Eq 4) as:

$$h\prime =\underset{event}{\int }wdt=\frac{-1}{\rho_{0}fL}\underset{event}{\int }\tau_{xs}dt+\frac{1}{L}\underset{event}{\int }Hv_{g}dt,$$ (5)

where for simplicity we have treated *L* as a constant, which is reasonable given that it is relatively constant year-round (i.e., when *N* tends to be large *H* is small and vice versa).

## Hydrodynamic Results

**Fig 3:**
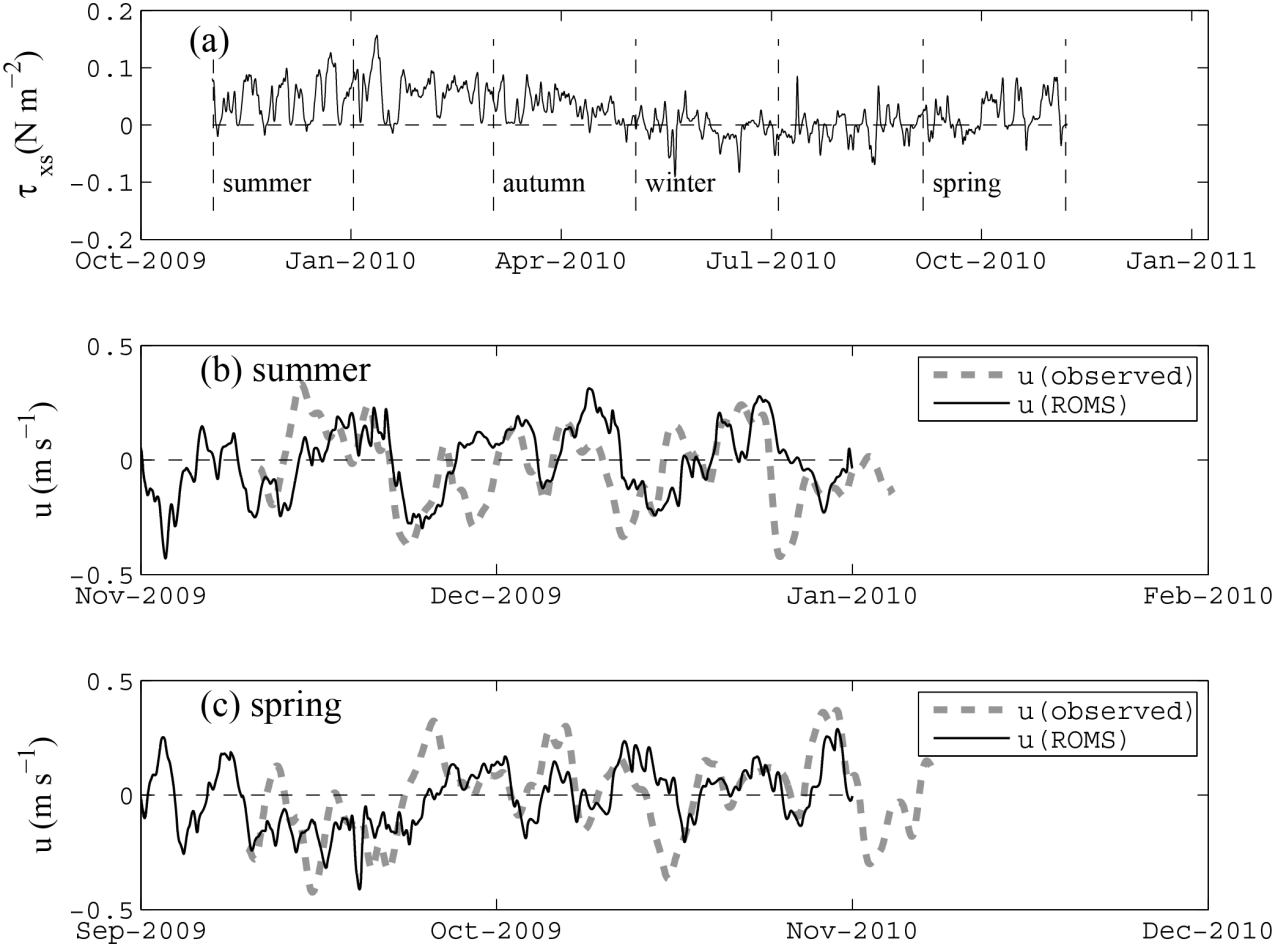
Comparison between the observed and modelled along-shelf currents. (a) Low-pass filtered along-shelf component of the wind stress (positive towards the northwest) over the year-long study period. (b) Comparison between the observed and modeled low-pass filtered along-shelf currents during the summer period, averaged over depths between 20–30 m. (c) Comparison of the observed and modeled low-pass filtered along-shelf currents during the spring period. The low-pass filter has a half-power period of 38 h to remove inertial oscillations, and in the case of the field data, the tides [30]. Note that the model results based on Xu et al. [18] do not include tides.

The ROMS model was previously evaluated in Xu et al. [18] using current and temperature profile data from a number of moorings deployed on the shelf near the North West Cape of Ningaloo Reef, specifically during the austral summer period (Nov 2009-Jan 2010). For this same period, we showed the modeled along-shelf currents at the M2 mooring at ~50 m depth (see location in Fig 1) with the observations (Fig 3b). In the present study, we also extended our model validation to include field observations described in Xu et al. [9] that focused on the austral spring period (Sept-Nov 2010) at the same mooring location (Fig 3c). The model performance in predicting the shelf currents was comparable between summer (model *skill* = 0.77, as reported in Xu et al. [18] where *skill* was defined by Willmott [29]) and spring (model *skill* = 0.73) at the M2 mooring. While there were no direct mooring observations in the other two seasons, comparisons of satellite SST observations with variability in the surface temperature fields modelled by ROMS showed good agreement (see Supporting Information, S2 Fig). Therefore, for these two intermediate periods when no mooring data were available, we expect that the model simulated the shelf ocean dynamics with comparable *skill*.

**Fig 4:**
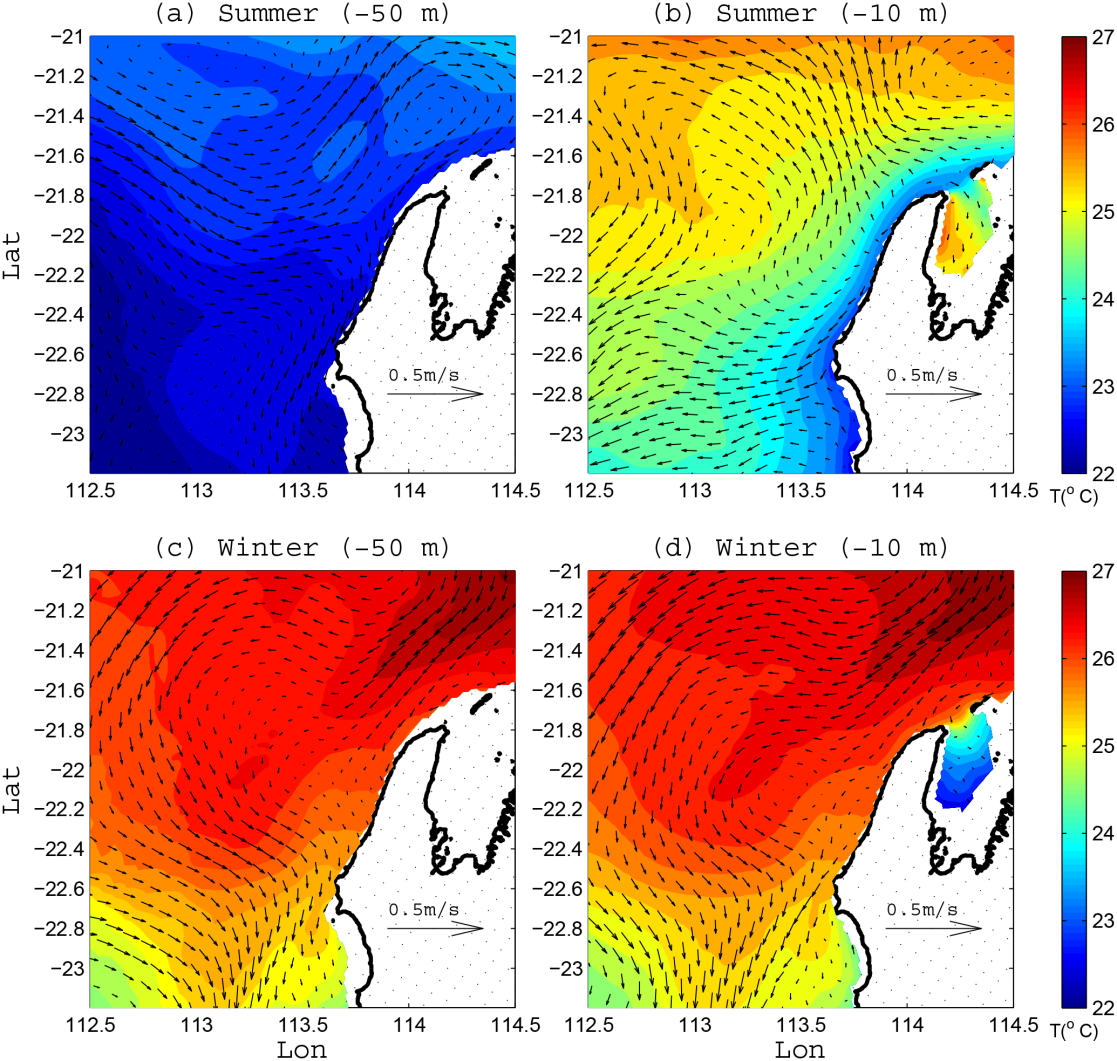
Modelled seasonally-averaged temperature (colormap) and current vector fields (a, c) at 50 m depth and (b, d) at 5 m depth. (a, b) show the average during the summer simulation between 1 Nov–30 Dec 2009, while (b, d) show the average during the winter simulation between 1 May–30 Jun 2010. White areas indicate land or those areas shallower than the plotted depth.

For the summer period, the mean velocity fields reveal distinct differences between the surface (5 m depth) and subsurface (50 m depth) flows: the subsurface flow was predominantly onshore (Fig 4a), while the surface flow was directed offshore (Fig 4b). The surface temperatures close to the coast were also lower than those offshore. These temperature and current field patterns are both consistent with coastal upwelling. Conversely, for the winter period, the mean velocity fields (Fig 4c and 4d) displayed similar patterns within both the surface and subsurface regions: a poleward, meandering along-shelf current at both depths and a poleward decrease in temperature. While results are not shown for the two transitional periods (i.e., autumn and spring), the flow patterns in these other seasons were intermediate between summer and winter. It is important to emphasize that while there are clear differences in the seasonally-averaged flow patterns in Fig 4, we show below that propagating mesoscale eddies and transient wind-driven coastal upwelling events were also present in the region, which added substantial higher frequency flow variability on this mean circulation.

## Lagrangian Particle Tracking Results

### 4.1 Scenario 1: Forward surface particle tracking (60-day duration)

**Fig 5:**
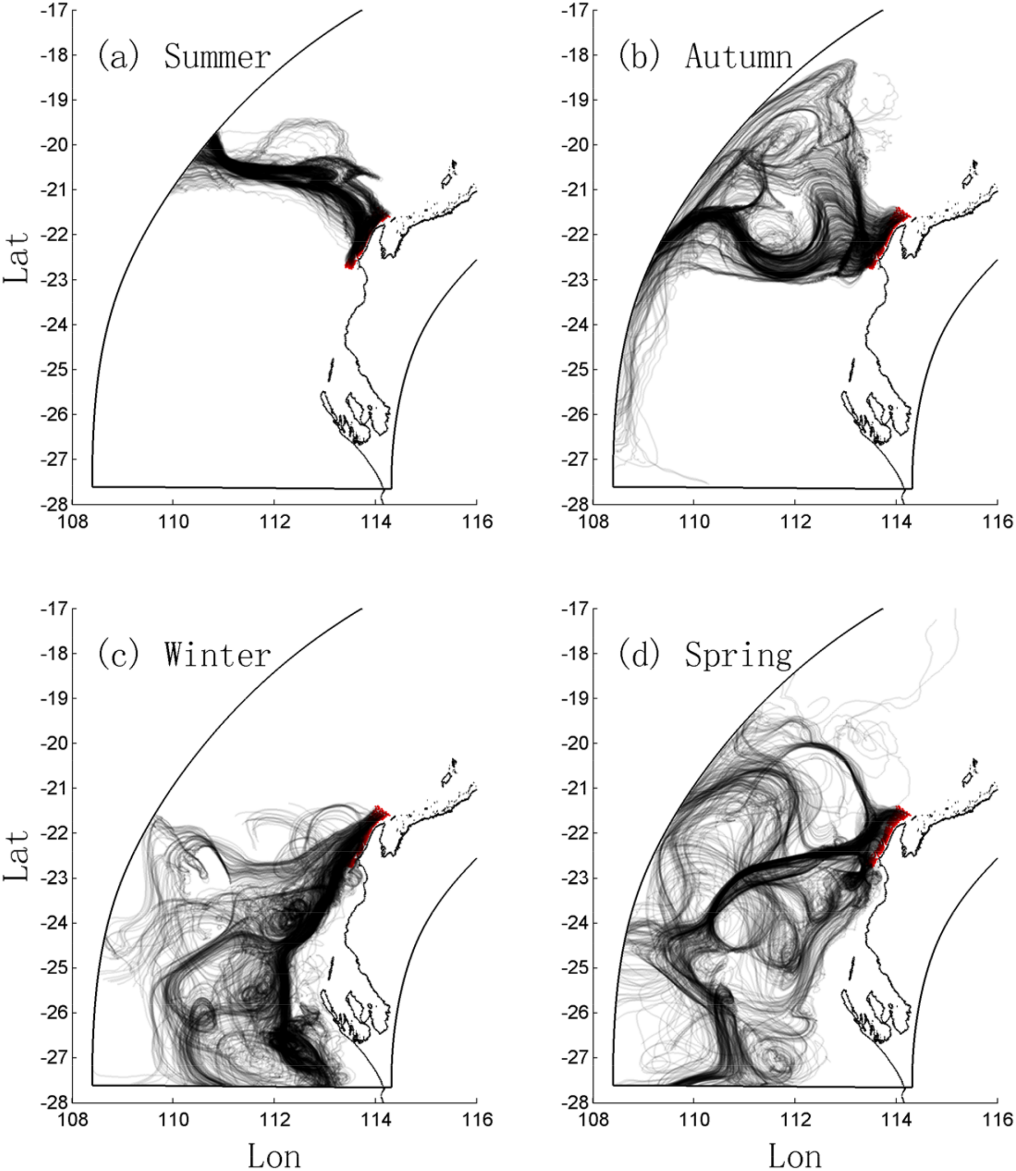
Particle trajectories from the forward-in-time tracking for Scenario 1. (a, b, c, d) the 60-day trajectories during summer (1 Nov 2009 to 1 Jan 2010), autumn (1 Mar 2010 to 1 May 2010), winter (1 May 2010 to 1 Jul 2010) and spring (1 Sep 2010 to 1 Nov 2010), respectively. Particles were released at the surface at locations indicated by the red dots.

The FITT results in Fig 5 show the fate of particles initially released near the coast at the surface during each seasonal period. In summer (Fig 5a), the prevailing upwelling-favorable wind resulted in the particles being advected offshore towards the northwest, with most eventually leaving the model domain. In autumn (Fig 5b), while there was still a surface transport of particles offshore (towards the northwest), due to the combination of the seasonal weaker upwelling-favorable winds and the influence of stronger offshore eddies, the offshore transport was weaker and more complex. In winter (Fig 5c), particles were moved poleward by the seasonally strong LC, often being entrained into large offshore eddies. In spring (Fig 5d) the transport pathways had some similarity to those observed in autumn. As shown in Appendix A, the random walk module had a negligible influence on these particle trajectories.

### 4.2 Scenario 2: Backward subsurface particle tracking (60-day duration)

**Fig 6:**
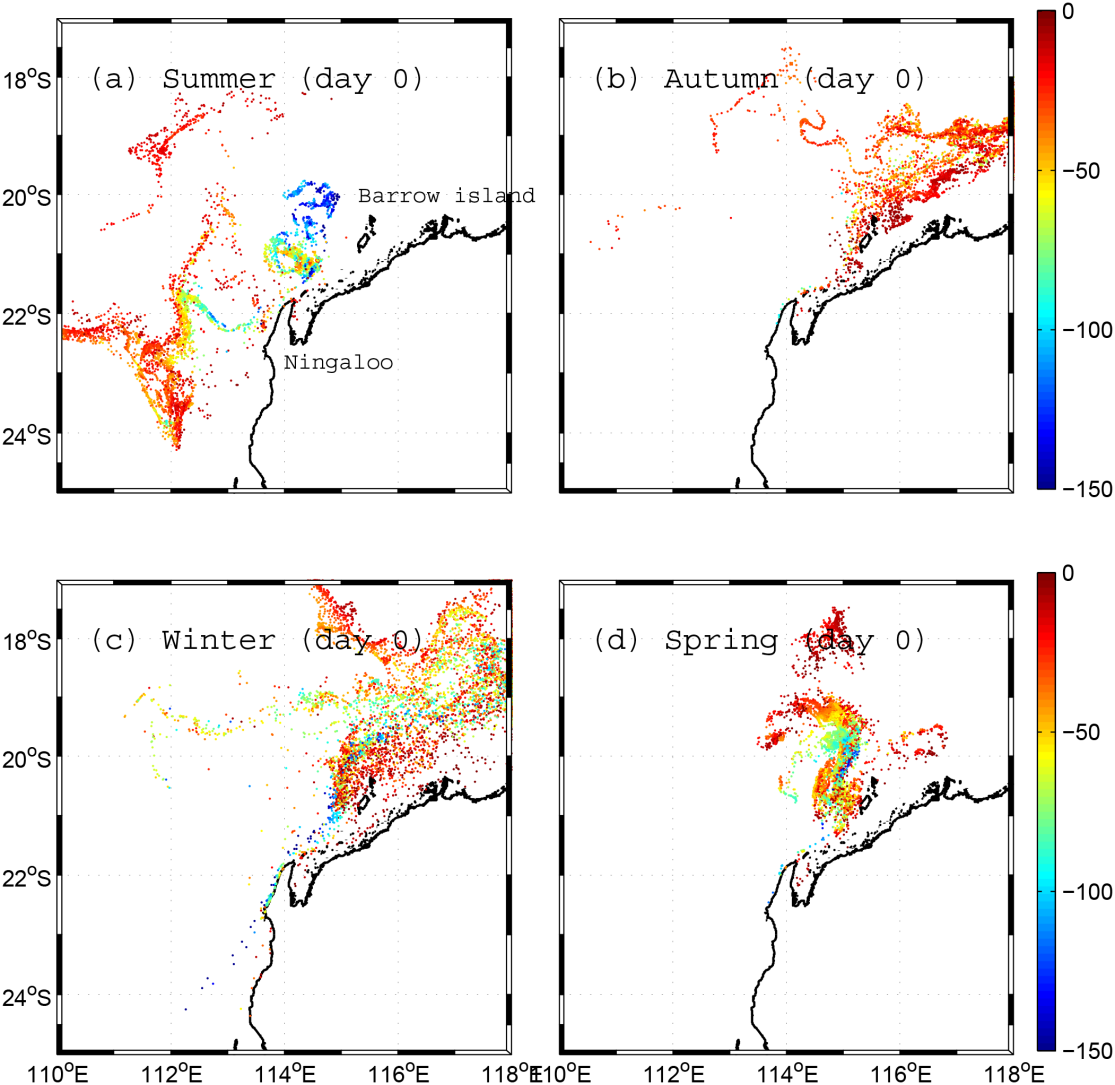
Source locations of particles transported to the Ningaloo shelf (initialized in PRZ1 zone in Fig 2b) for Scenario 2. (a, b, c, d) the source location of particles after two months (day 0) of BITT simulation during summer, autumn, winter and spring, respectively. The colors denote the source depth of the particles in meters.

The source regions predicted by the 60-day BITT simulations in Scenario 2 study are shown in Fig 6a–6d. In summer, particles originated from two main sources (Fig 6a). A relatively shallow source (20–60 m depth range) was located to the west, accounting for around 70% of particles that reached Ningaloo; whereas a deeper source (>60 m) was located to the north of Ningaloo, accounting for most of the remainder. During the other seasons, the source region was always to the northeast of Ningaloo, at depths ranging from relatively shallow (<50 m) in autumn to deeper but more variable (up to 150 m) in winter and spring (Fig 6b–6d).

**Fig 7:**
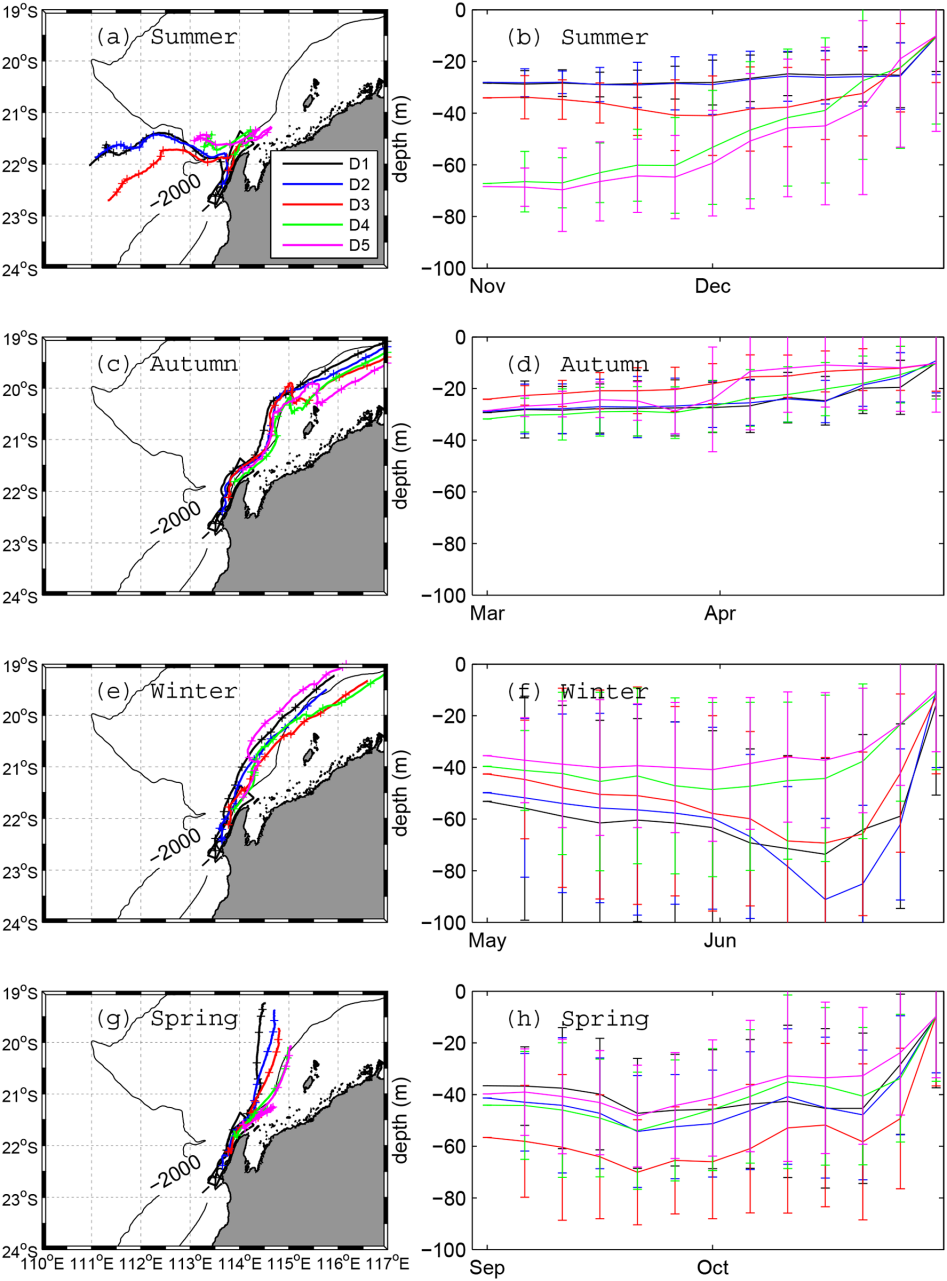
The mean particle transport pathways and the corresponding mean vertical location as a function of time for the BITT simulation in Scenario 2 study. (a, c, e, g) show the mean pathways of particles that reach each sub-domain D1, D2, D3, D4 and D5 in corresponding season. (b, d, f, h) show the mean and standard deviation (error bars) of the depth of the particles every 5 days.

The mean transport pathways of water that reached the five coastal sub-domains are shown in Fig 7 (from south to north termed D1, D2, D3, D4 and D5, see Fig 2). In summer (Fig 7a and 7b), particles were transported by an eastward flow, but with particles that reached the northern sub-domains (i.e., D4 and D5) originating from deeper sources than those reaching the southern sub-domains (i.e. D1–D3). In autumn, the particles originated from similar depths across all sub-domains and were transported by a southwestward flow (Fig 7c and 7d). During winter (Fig 7e and 7f), the pathways were similar to autumn, but particles originated from a much deeper source. During the spring season (Fig 7g and 7h), again only one primary southward transport pathway was identified; however, these particles originated from a region slightly further to the west than during autumn and winter.

In addition, for Scenario 2 we also conducted a sensitivity analysis of the model results to the exact initialization (or release) time of particles (see Appendix B for details). Due to the highly transient nature of the flows, there was indeed some weak sensitivity to this initialization time, but most importantly the transport paths described were still found to be representative and consistent within each season. Moreover, while the focus of the Scenario 2 analysis was on identifying the sources of the particles interacting directly with the reef, we also conducted simulations to investigate the origin of particles reaching deeper water (i.e. 50 m depth) on the Ningaloo shelf (see Supporting Information, S3 Fig). For the majority of the year, these transport pathways were similar to Fig 7, except in spring when two different onshore transport paths were observed. In general, these pathways indicate that particles reaching Ningaloo Reef originate from the northeast for most of the year; however, during summer there is an additional source from the west. Throughout the year, there was no evidence of any southern source.

### 4.3 Scenario 3: Backward subsurface particle tracking (10-day duration)

**Fig 8:**
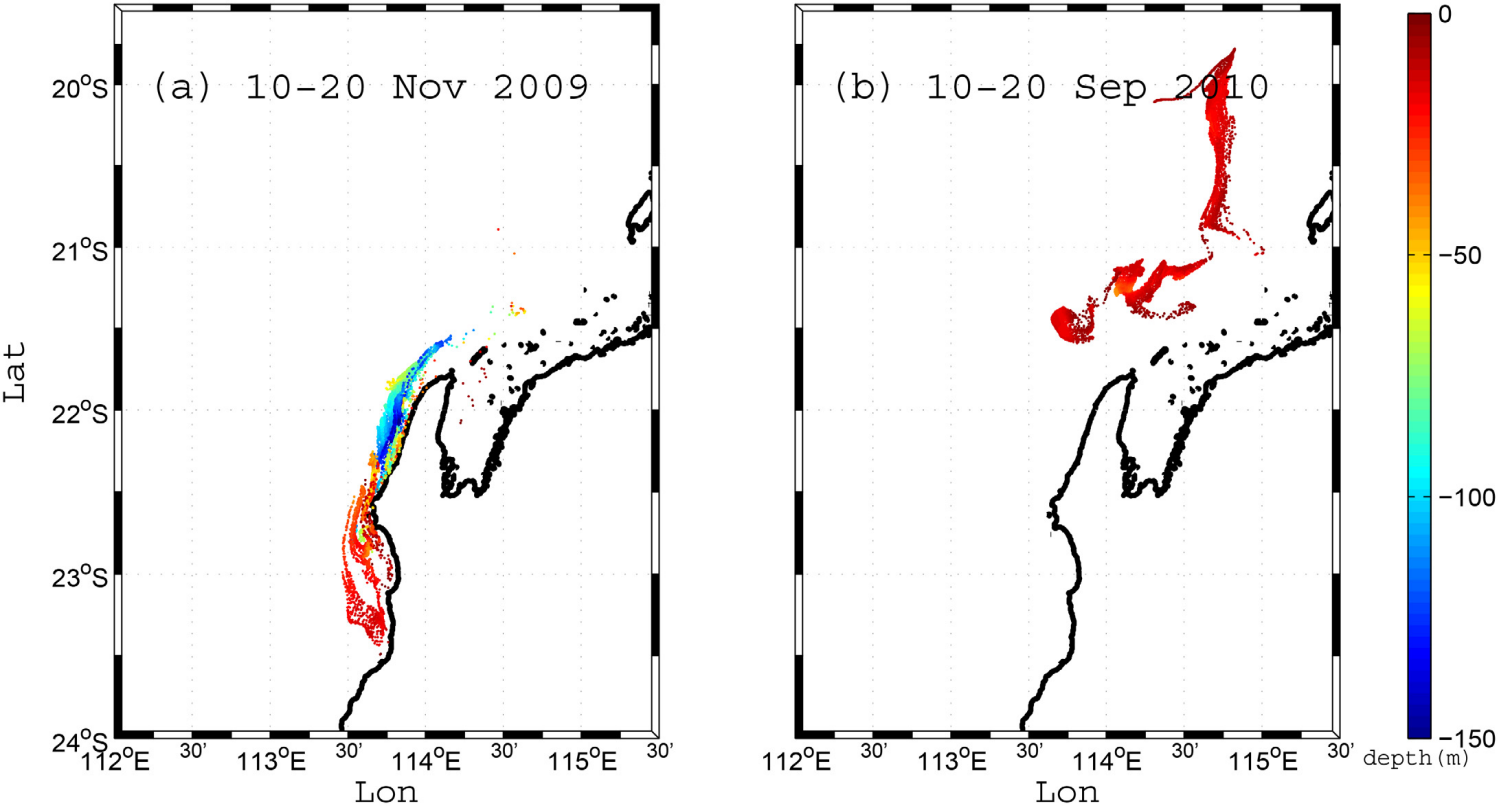
Backward particle tracking results for two distinct 10-day periods for Scenario 3, with the color denoting the source depth (m). (a) The source region of particles for the 10–20 Nov 2009 simulation. (b) The source region of particles for the 10–20 Sep simulation. In both cases the particles were initialized in PRZ2 in Fig 2.

The Scenario 3 simulations focused on identifying the higher frequency variability in source locations and depths using 10-day BITT simulations that approximately coincide with the dominant 1–2 week synoptic wind forcing period in the region [1]. Example simulations for two contrasting shelf circulation events are shown in Fig 8. During the first period (10–20 Nov 2009), the winds were northward and upwelling favorable and thus the Ningaloo coastal region contained a large portion (~37%) of water originating from depths >100 m (Fig 8a). Conversely, during the period from 10–20 Sep 2010 when winds were very weak, the water was sourced from northeast of the Ningaloo Peninsula at only shallow depths (typically <30 m, Fig 8b).

**Fig 9:**
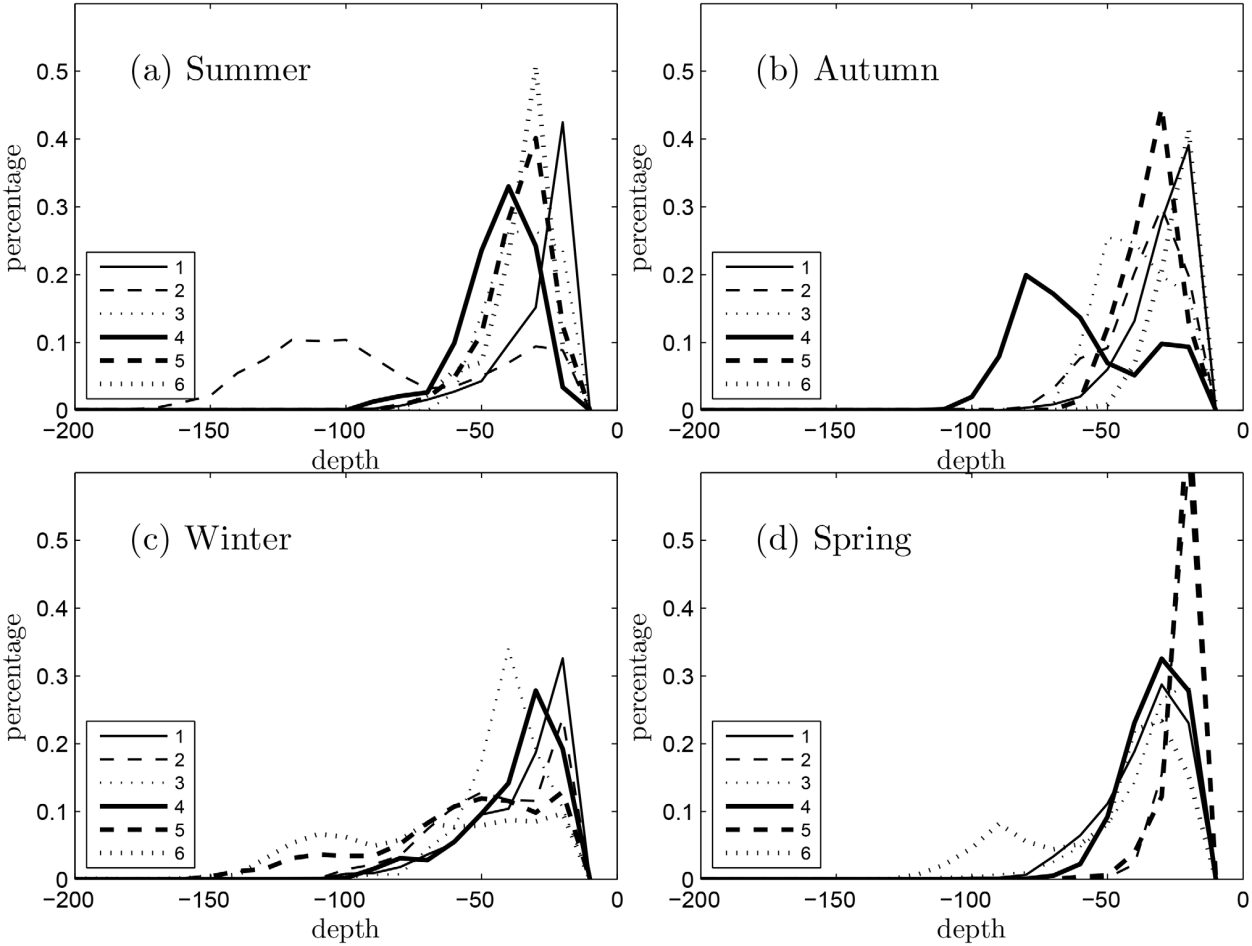
Histograms showing the statistical distribution of the particle source depths obtained from the 10-day BITT simulations during each season (Scenario 3). The coloured lines (i.e. 1, 2, 3, 4, 5, 6) denote results from each 10-day simulation within a season.

In Fig 9, we quantified the statistical distribution of the particle source depths that reached the coastal region off Ningaloo Reef (region PRZ2 in Fig 2) for each of the six 10-day simulations conducted within each seasonal period in Scenario 3. The results revealed that particles that reached the surface waters adjacent to Ningaloo almost entirely originated from depths shallower than 100 m (and mostly from <50 m) throughout the year. These source depth histograms also tended to be very similar during three of the four seasons (summer, autumn and spring), with most water sourced from 30–50 m depth. However, during these three seasons there were also some anomalous events, e.g., run 2 in the summer and run 4 in autumn, where a large portion of the particles were sourced from >50 m depth and reached depths up to 150 m; these correspond to very strong upwelling events, e.g., the 10–20 Nov 2009 upwelling event (see Fig 8a and [9, 18] for details). The winter season was most distinct, usually with a more uniform source distribution over a broad range of depths, occasionally extending down to over 100 m.

**Fig 10:**
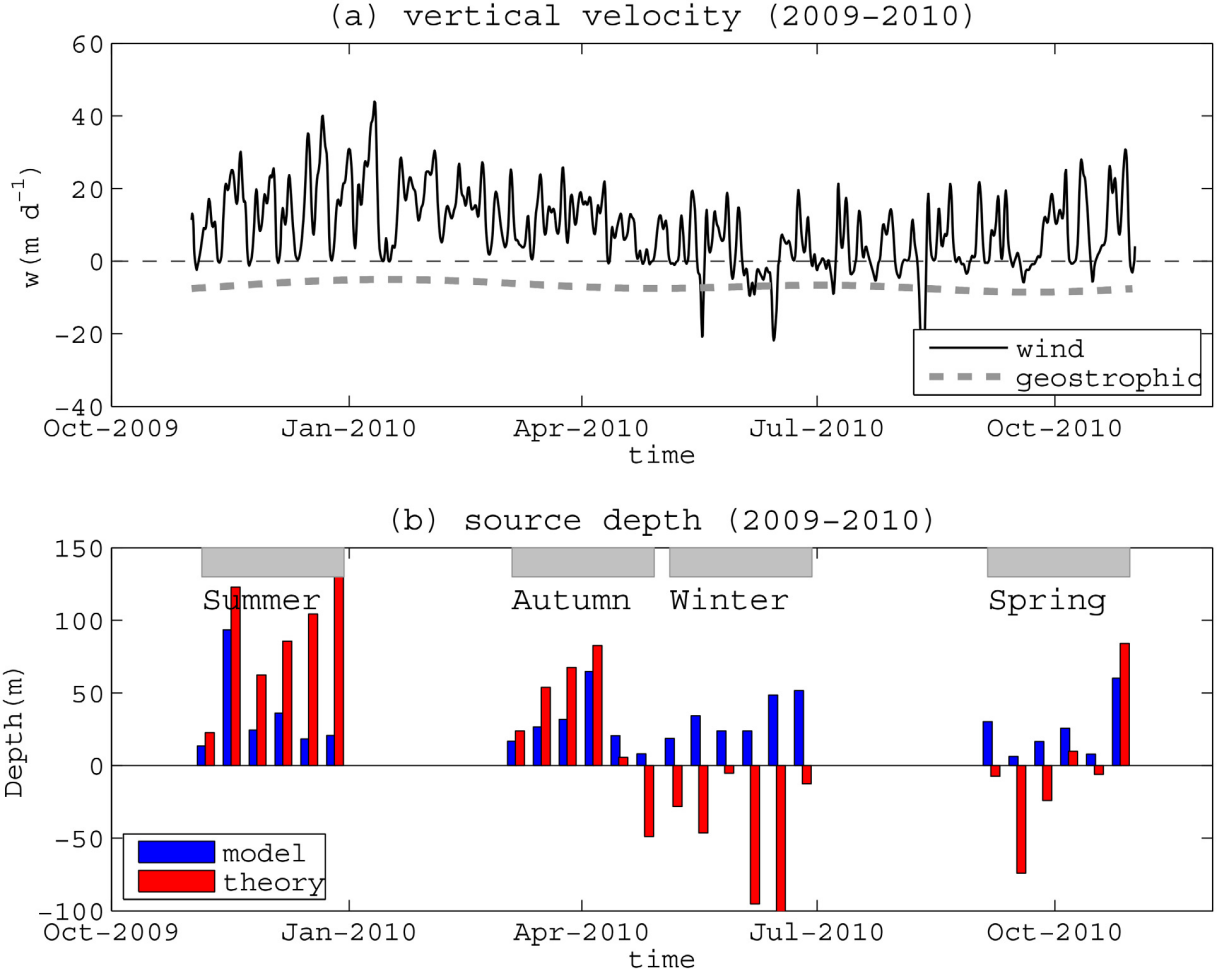
Time series of the theoretical vertical velocities (*w*) predicted from (Eq 4) and their integrated vertical displacements (*h’*) from (Eq 5). (a) Predictions of the vertical velocity (m day^-1^) from the along-shelf winds (thin solid line, term 1 in (Eq 4)) and geostrophic transport (thick dash line, term 2 in (Eq 4)). (b) Comparison of the simulated vertical displacement distances of particles initialized in PRZ2 in Fig 2 (blue bars) with the theoretically predicted values from (Eq 5) (red bars). Each bar represents the domain (PRZ2) averaged vertical displacement of particles over each individual 10-day simulation period, with each season highlighted by the grey boxes.

Using these Scenario 3 simulation results, we also compared the vertical particle excursion velocities *w* predicted from the numerical model with analytical upwelling theory using (Eq 4) (Fig 10). The decomposition of the terms that contribute to (Eq 4) in Fig 10a reveals that the Ekman transport term was generally predicted to be positive (consistent with the dominantly upwelling-favorable winds year round), but was also highly transient. Conversely, the geostrophic velocity term is predicted to be responsible for a near-constant downward velocity of ~7 m day^-1^ (Fig 10a). Using these values of *w*, we applied (Eq 5) to predict the source depth *h’* of water that reached the Ningaloo coastal region and compared these depths to those in the particle tracking simulations. Throughout the summer, the wind-induced transport contribution was strong enough to move particles up towards the surface, although the modelled vertical particle excursions were generally smaller than predicted from the theory (Fig 9b). In autumn, the modelled vertical particle excursions generally agreed well with the theory. However, during both winter and the initial part of spring, when the coastal winds were often weaker and directionally-variable, the analytical model ((Eq 4)) agreed poorly with the modelled vertical particle excursions. During this period, (Eq 4) predicted that strong downwelling conditions should prevail (indicated by the negative source depths), whereas the numerical model predicted some weak upward transport. In general, the analytical coastal upwelling model given by (Eq 4) did not appear to provide a complete description of the vertical motion of particles along Ningaloo Reef, which we discussed further in Section 5 below.

## Discussion

### 5.1 Seasonal variability in connectivity pathways

The results from the longer-duration particle tracking simulations (Scenarios 1 and 2) provided insight into how the regional-scale transport pathways that are responsible for exchanging water to and from Ningaloo Reef can seasonally vary across the region. During the summer period, the source region was influenced by both local coastal wind-driven upwelling events and a subsurface flow field that was remotely-forced by large-scale pressure gradients operating in the region. Water parcels were initially advected in the subsurface region before being transported to the surface by localised coastal upwelling close to the Ningaloo coast (typically only within a few Rossby radii or O(10 km)). During this summer period, particles were transported either from offshore or from a nearby northern region, suggesting two potential onshore transport mechanisms: a bottom onshore-directed flow associated with coastal upwelling (deeper source) and an onshore geostrophic flow (shallower source). During the autumn period, despite the winds often being of comparable magnitude to the summer, particles instead originated from the northeast region of Australia’s NWS. This confirms the presence of a weak seasonal (autumn-winter) poleward flowing current on the NWS (i.e., the so-called Holloway Current) that was originally proposed by Holloway [7], and reinforced by recent field observations reported by Ridgway et al. [31]. While upwelling events along Ningaloo were observed to occur year-round, during the winter period when the wind was either weak or exhibited large variability, it was very rare to observe any persistent coastal upwelling. During winter, the particles were sourced from a much broader range of depths (up to 150 m), also consistent with the deepening of the surface mixed layer depth during the winter months in this region.

The present study focused on assessing how the transport pathways to/from Ningaloo Reef varied in different seasonal periods during 2009–2010, and while inter-annual variability in the regional ocean dynamics could modify these pathways on a year-by-year basis, we still expect these pathways to be generally representative of the longer-term seasonal variability that occurs in the region. Lowe et al. [1] assessed the seasonal variability in the shelf circulation off Ningaloo Reef using long-term mooring records, and observed only relatively weak inter-annual variability in the seasonally-averaged transport, including the same general seasonal trends that we observed in the present study. Moreover, based on the Southern Oscillation Index (SOI), this 2009–2010 study focused on a neutral period in the El Niño-Southern Oscillation (ENSO) cycle, occurring within a transition between El Niño to La Niña conditions [32]. Thus, while it is impractical to conduct this detailed particle tracking analysis over long (i.e., decadal) periods, we do not expect these transport pathways to differ substantially between years, although future work would need to confirm how potential climate variability modifies these dynamics.

### 5.2 Impact of geostrophic flow, mesoscale eddies and buoyancy on vertical transport

The shorter-duration (10 day) Scenario 3 simulations provided insight into the processes that contribute to vertical transport along the Ningaloo shelf, including the role of different forcing mechanisms. A simple analytical model for predicting coastal upwelling / downwelling was proposed by Marchesiello et al. [28], which accounts for the effect of wind-driven surface Ekman transport in the presence of an interior geostrophic flow (i.e., (Eq 4)). Following a similar approach to Rossi et al. [25], we used (Eq 4) to predict upwelling velocities from both the measured winds and climatological estimates of variability in the cross-shelf geostrophic transport from CARS09, and compared the results with those predicted using our three-dimensional region ocean circulation model. Our results in Section 4.3 revealed that this analytical model had variable performance (Fig 10), occasionally reproducing the upwelling transport well (i.e. during part of the summer and much of the autumn period), but generally failing to predict both the correct magnitude of the transport and even its correct direction (i.e., upwelling versus downwelling) during the winter and early-spring periods. We note that Xu et al. [9] subtracted the interior geostrophic transport from a number of mooring observations along Ningaloo, and found that the theoretical Ekman transport (i.e. the first term (Eq 4)) agreed well with field observations. This indicates that the major discrepancy with the analytical model is the overly-simplified representation of the upwelling / downwelling response to the barotropic cross-shelf flow (i.e. the second term in (Eq 4)) estimated from climatological (monthy-mean) data (i.e. CARS09) and hence assuming a quasi-steady cross-shelf geostrophic transport. Below we discuss the various mechanisms that likely contribute to the observed discrepancy.

One of the most important contributions missing in (Eq 4) is from mesoscale eddies, which have been observed to dominate the circulation along Ningaloo when they regularly encroach onto the shelf [9, 14]. These eddies are prevalent features along WA, with the Leeuwin Current displaying the highest eddy kinetic energy levels among all mid-latitude eastern boundary currents [10]. As a consequence, the total local geostrophic transport would be comprised of contributions from both a mean geostrophic flow and mesoscale eddies, which in practice can be difficult to separate during individual events. Examples of the impact of mesoscale eddies on the shelf circulation of Ningaloo have been regularly observed in both satellite images and mooring measurements in this region; for example, in Xu et al. [9] (see Fig 15 and Section 5.3 in that paper) and also in Rossi et al. [14]. Therefore, the presence of mesoscale eddies encroaching on the Ningaloo shelf should have a significant impact on the dynamics of coastal upwelling.

**Fig 11:**
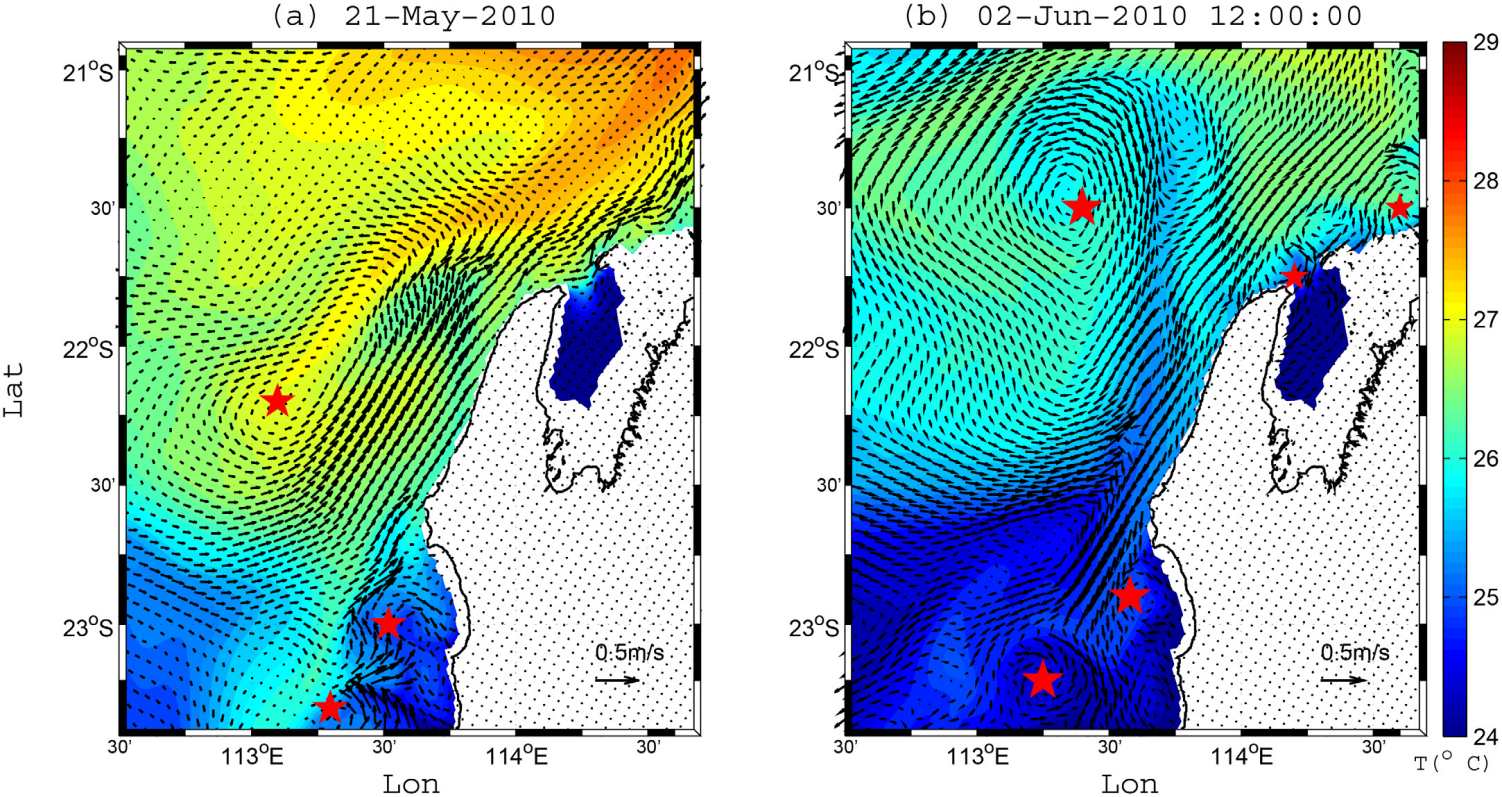
Modelled velocity vectors and temperature field at 1 m below the sea surface on (a) 21 May 2010 and (b) 2 Jun 2010. The center of eddies are denoted by red stars for both periods.

In the winter period (including adjacent months) when coastal upwelling was rarely observed, these eddies appeared to be particularly important in driving vertical transport that was not predicted by (Eq 5) (Fig 10). Thus, our model results suggest particles can be advected substantially upward or downward by eddying motions, often occurring at the boundaries between cold and warm water masses (Fig 11). These eddies range in size from mesoscale (10s km), where Coriolis forces dominate, to sub-mesoscale where both Coriolis and centrifugal forces are important (i.e. some eddies are highlighted by the red stars in Fig 11). As shown in Fig 11 during two winter periods, eddies that encroach towards Ningaloo Reef can substantially alter the shelf circulation. Klein et al. [33] provided a general review of the vertical exchange of material in the ocean associated with mesoscale and sub-mesoscale eddies, and while focusing on the open ocean, they demonstrate how important eddies can be to localized vertical transport. In shelf regions, eddies have also been recognized for the important role they can play in vertical transport. For instance, Bassin et al. [34] showed that coastal eddies can contribute a major source of vertical nutrient flux to the inner shelf region of the California shelf. For our study region, the relatively weak stratification in winter also appears to enhance vertical displacements of water from these eddies. This suggests that along coasts with consistent downwelling (e.g., along WA) transient eddies can still drive substantial upward transport on the shelf, especially during seasons when the stratification is weak.

Interactions between buoyancy forces and shelf slopes can also complicate the response of coastal upwelling predicted from the simple analytical model [35–39]. Buoyancy arrest effects can modify the cross-shelf transport and vertical transport structure, and hence suppress vertical transport. Both the present model results and our prior field observations [9] indicate that this buoyancy arrest effect can be strong during the spring, summer and autumn seasons, when the Burger number (*B = NH/f*) consistently exceeds 2 along the majority of the Ningaloo shelf. For cases with large Burger numbers, the flow within the bottom boundary layer contributes to strengthening the vertical density gradient, and the cross-shelf flow can be ‘shut down’ after a short adjustment time [35–37] (approximately 1 day during summertime on the Ningaloo shelf, which equates to a small excursion length of < 5 km on the shelf), as was shown in our previous observations at Ningaloo [9, 18]. The strong buoyancy gradient across the shelf can thus substantially reduce onshore bottom boundary layer flow, while still allowing for the development of onshore geostrophic flow in the interior of the water column. This is consistent with the two main water sources observed in our particle tracking results, i.e. a deep source associated with bottom boundary layer transport and a shallower source due to onshore geostrophic transport. The idealized upwelling model ((Eq 4)), will only predict the instantaneous vertical transport velocity just below the surface boundary layer, so it is clear that a simple integration of this velocity can overestimate the overall source depth by ignoring the actual vertical flow structure. This is also the reason why Lagrangian particle tracking can give more reasonable predictions of true vertical migration distances, since it continuously follows the position of particles as they move through this spatially-varying flow field.

### 5.3 Implications for nutrient supply to Ningaloo Reef

Our connectivity study has important implications for identifying the regional transport pathways and source waters that supply nutrients to Ningaloo Reef in both dissolved and particulate forms. Based on phytoplankton grazing rates by shallow reef communities measured on a portion of the ~300 km long reef, Wyatt et al. [13] proposed that the oceanic supply of nitrogen to the reef that is associated with phytoplankton must be drawn from a large offshore ‘ocean catchment’ in the region. From the seasonal transport pathways identified in this study (e.g. Figs 6 and 7), there is clearly the potential for the regional ocean circulation to supply this reef with particulate nutrients originating from source regions that are remote from the reef itself.

**Fig 12:**
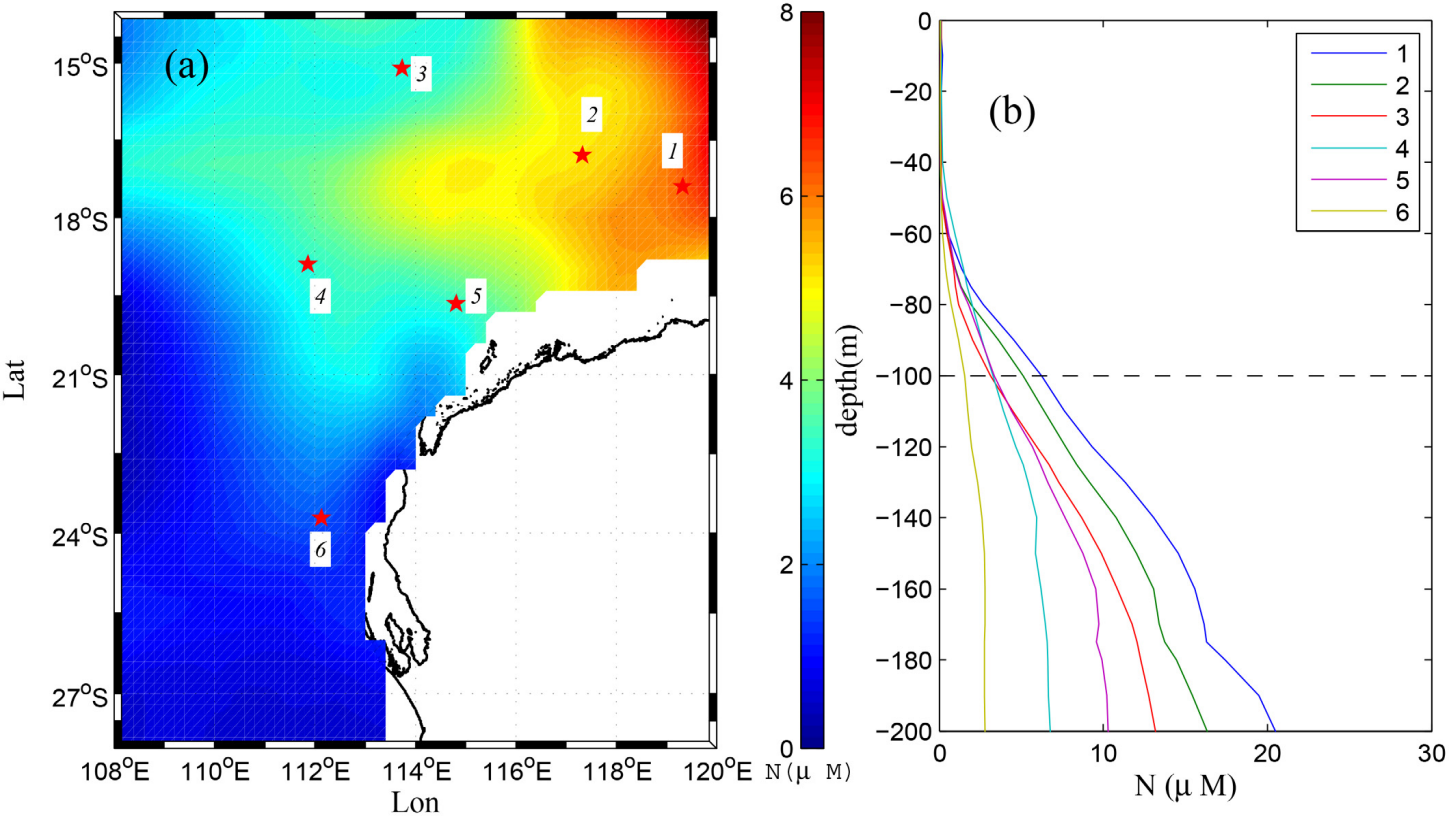
A climatological snapshot of nutrient (as nitrate) distribution taken from CSIRO Atlas of Regional Seas (CARS) for 1 Nov [41]. (a) the horizontal distribution of nitrate (NO_3_, in units of uM) at 100 m depth as denoted by the back dash line in (b); (b) the vertical profiles of nitrate concentration taken at six sites highlighted by the red stars in (a).

In addition, examination of the seasonally and spatially-varying dissolved nitrogen (nitrate, NO_3_) concentrations from historical archives (CSIRO Atlas of Regional Seas, CARS 2009) shows very low concentrations (~0 μM) in the upper 50 m of the water column throughout the region (Fig 12). However, concentrations increase sharply below 50 m, and tend to also increase towards the northeast, reaching ~10 μM at some northern locations at 100 m depth (Fig 12). According to these regional-scale NO_3_ patterns and our estimation of the seasonal source water locations and depths (Section 4.2), it is apparent that water at the Ningaloo coast during most of the year (Mar-Oct) is on average sourced from the surface waters (<50 m depth) where NO_3_ concentrations are very low. Some coastal upwelling may bring up deeper (NO_3_ rich) water from 50–100 m depth to the surface in spring and summer; however, the strengthening of the poleward flow on the NWS would also advect higher NO_3_ concentration water southward towards Ningaloo Reef during the late-summer and autumn periods. Although we acknowledge that dissolved nutrients such as NO_3_ do not behave as passive tracers, as they are taken up and transformed by planktonic communities, the results suggest that horizontal advection of nutrients may be an important source of nutrients to Ningaloo Reef during these seasons. Only during occasional major upwelling events (especially in summer), would coastal upwelling be able to bring up large volumes of deep water (100–150 m) with high NO_3_ concentrations (> 5μM). Nevertheless, upwelling events would regularly deliver moderate-depth water (~50–80 m) towards the surface, where nitrate concentrations are typically ~1 μM and are thus still far greater than background surface water concentrations. This magnitude of NO_3_ variability (up to ~1 μM) associated with typical upwelling events at Ningaloo compares well with the observations from Wyatt et al. [40], which attributed increases in NO_3_ concentrations from background levels of <0.3 μM to ~1 μM to periods of persistent upwelling favorable winds that most often occur in summer. However, our model results showed there was only limited coastal upwelling in the winter; instead deep and hence nutrient rich water was transported upwards by coastal eddies. Thus, transient eddies propagating through this region can also be an important mechanism in supplying nutrients to the shallow waters of Ningaloo Reef.

## Conclusions

In this study we investigated the ocean transport pathways surrounding Ningaloo Reef during different seasonal periods, and how they are influenced by transient upwelling, the Leeuwin Current and energetic eddy fields in the region. Overall, two main source regions for water that interacts with Ningaloo Reef were identified: water from the northeast on Australia’s North West Shelf (in all seasons); and water from the west and offshore of the Ningaloo shelf (particularly in summer). The poleward circulation associated with the Leeuwin Current dominated horizontal transport during most of the year, consistent with the predominant southward along-shelf trajectories of particles that originated from the northeast. The offshore water source was generally only significant during the summer period, when the Leeuwin Current was comparatively weak and was located further offshore. The vertical position of source waters were influenced by transient coastal upwelling that was found to be important on the Ningaloo shelf during the spring, summer and autumn periods. Driven by local wind variability and also influenced by the prevailing onshore geostrophic transport, coastal upwelling episodically brought subsurface water (typically from 100 m depth, but occasionally from 150 m during major upwelling events) towards the surface along the Ningaloo Reef. This persistent onshore geostrophic flow had an important influence on the development of coastal upwelling year-round, while transient eddies encroaching on the shelf also episodically contributed to the vertical advection of deep water to the reef, particularly in winter when the surface stratification was weakest. Overall, the results reveal how the complex ocean dynamics of the region determine the material transport pathways to Ningaloo Reef, which has important implications for understanding the mechanisms responsible for supplying nutrients to the reef that are required to support its high productivity.

## Appendix A. Sensitivity of the Random Displacement Module

**Fig 13:**
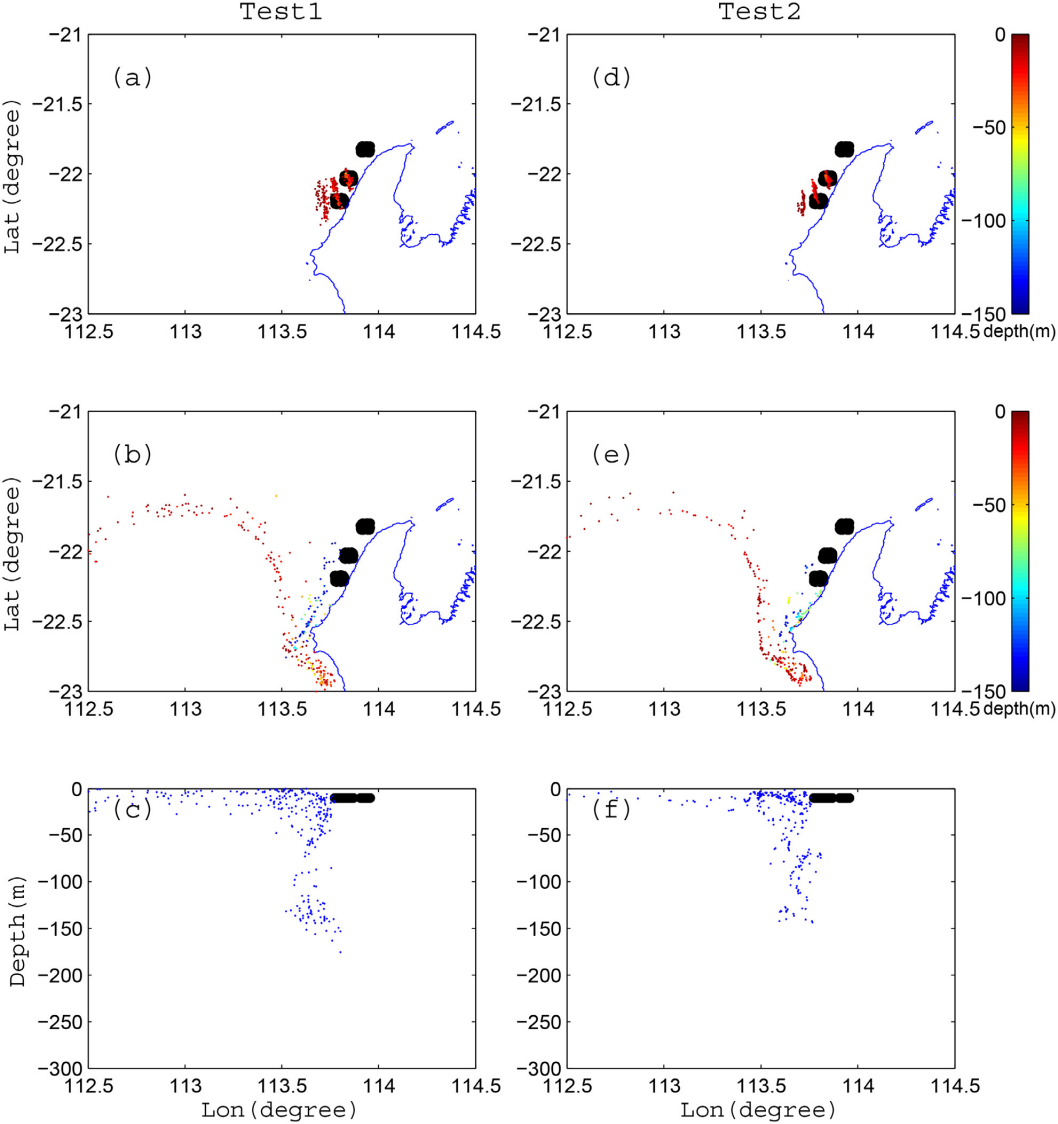
A comparison of particle tracking results with both advection and particle diffusion included (Test 1, left column) and with only advection included (Test 2, right column). (a, d) show maps of the particle positions after 2 days of simulation, and (b, d) show the particle positions after 10 days of simulation. (c, f) show the longitude and vertical positions of particles in Test 1 and Test 2, respectively, after 10 days of simulation. The colorbars in (a, b, d, e) denote the vertical position of particles, while the black dots denote the initial position of particles at 10 m depth. In (c, f), the black dots denote the initial particle positions, while the blue dots denote their final position after 10 days of simulation.

While the random displacement model that is implemented in LTRANS is considered an effective tool for describing turbulent particle diffusion [23, 24], we initially conducted sensitivity tests to evaluate the relative importance of diffusion versus advection in determining the main particle trajectories. Two sets of FITT simulations were conducted where a total of 300 particles were initialized on 1 Nov 2009 at three sites (100 particles at each) along the Ningaloo coast at 10 m depth (Test 1, see Fig 13a, 13b and 13c). In Test 1, the model included both vertical and horizontal turbulent diffusion (i.e. using random displacement module) and was run forward-in-time for 10 days. In Test 2, we turned off the random displacement module and ran an advection only simulation for the same period (Test 2, see Fig 13d, 13e and 13f). For both test runs, we initialized particles randomly over a 0.05 degree square box (see the black cloud of particles in Fig 13a, 13b, 13d and 13e) surrounding the release sites. From Test 1, the particles were transported initially south and then back to north, and separated into two main horizontal branches: a shallow offshore branch and a deep nearshore branch (see Fig 13b). In Test 2, two very similar branches were found (compare Fig 13b and 13e). Similarly, when comparing the final vertical particle distributions in Fig 13c and 13f there were only minimal differences in the final vertical distribution of particles. Collectively these results indicate that, although turbulent diffusion is not entirely negligible, advection clearly plays a much more dominant role in determining the particle transport pathways. As a consequence, in our BITT model (where diffusive processes cannot be reversed) we can justify disabling the random displacement module, as it will only have a minimal influence on the particle trajectories, particularly when averaging over a large number of particles.

## Appendix B. Sensitivity of the Transport Pathways to Particle Initialization Time

**Fig 14:**
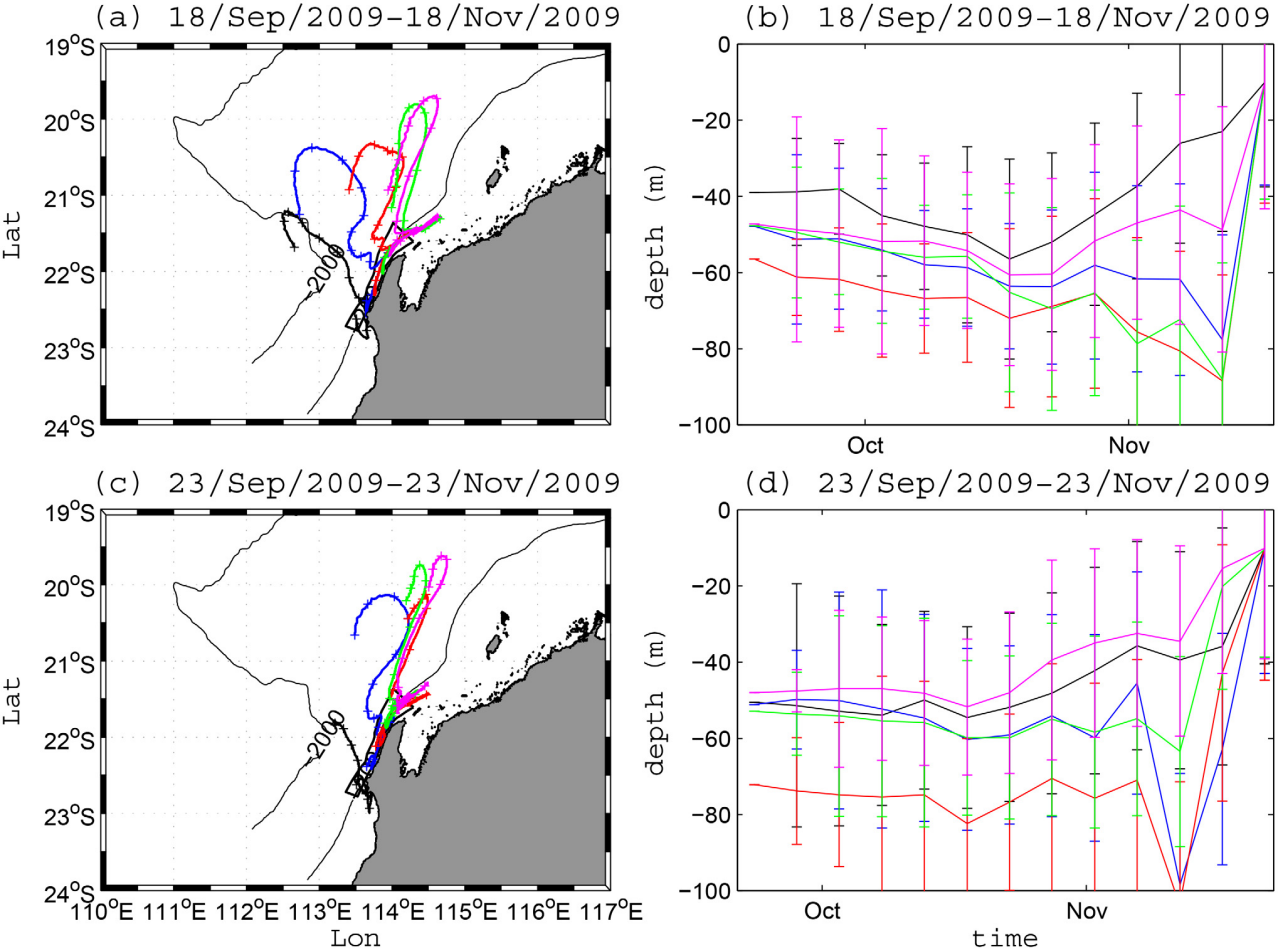
The mean particle transport pathways and the corresponding mean vertical position as a function of time. (a, c) show the mean particle pathways initialized at 10 m depth for each sub-domain, i.e. D1, D2, D3, D4 and D5 between 19 Sep–18 Nov 2009, and between 24 Sep–23 Nov 2009, respectively. In (b, d), the time series show the average and standard deviation (error bars) of the vertical location of the particles every 5 days.

To investigate the sensitivity of particle trajectories to their initialization time, we conducted two test simulations as part of Scenario 2 that were initialized on 18 Nov 2009 and at 23 Nov 2009, respectively, and were then run backward in time for 60 days (Fig 14). These periods were chosen given that the shelf circulation was substantially different during each initialization day (see Xu et al. [12]), transitioning from downwelling conditions on 18 Nov to upwelling conditions on 23 Nov. Both simulations show similar southward and onshore transport pathways (Fig 14a and 14c). The small differences were due to the strong upwelling on 23 Nov 2009, when relatively large vertical excursion of the particles occurred. There was a larger vertical transport during this event, when particles were transported from ~80 m towards the surface in Fig 14b and from ~100 m in Fig 14d. The transport pathways can therefore be weakly sensitive to the particle initialization time on relatively short (i.e. weekly) time scales, since this can depend on the timing of wind events (or upwelling events) that vary on similar time-scales. However, when assessing the longer-term transport pathways (i.e., over monthly time-scales), the results were not sensitive to the particular initialization time.

## Supporting Information

S1 Fig Comparison of both forward particle tracking with backward particle tracking (without turbulent diffusion). (a) shows the initial positions of all particles at day 0; (b) shows the final positions of all particles at day 5; (c) shows the positions of particles at day 0 after 5 days of backward particle tracking from (b).
(PDF)

S2 Fig Time series of the satellite SST from GHRSST and the modelled SST from the hydrodynamic simulations at M5 mooring site during Nov 2009 and Nov 2010. The bold dash line is the satellite SST, and the thin solid line is the modelled SST.
(PDF)

S3 Fig The mean particle transport pathways and the corresponding vertical particle migrations as a function of time, for each sub-domain i.e. D1, D2, D3, D4 and D5. (a, c, e, g) show the mean pathways of particles initialized on the Ningaloo shelf at 50 m depth during summer, autumn, winter and spring, respectively; (b, d, f, h) show time series of the average and standard deviation (error bars) of the depth of the particles every 5 days.
(PDF)
