# Supplementary figures and images for: Ocean Transport Pathways to a World Heritage Fringing Coral Reef: Ningaloo Reef, Western Australia
Source: PLoS One. 2016 Jan 20;11(1):e0145822. doi: 10.1371/journal.pone.0145822 (PMC4720280; doi:10.1371/journal.pone.0145822)

Ningaloo day0

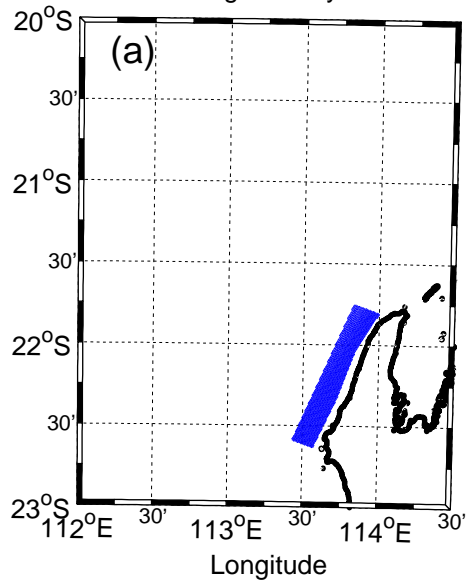

Ningaloo day5

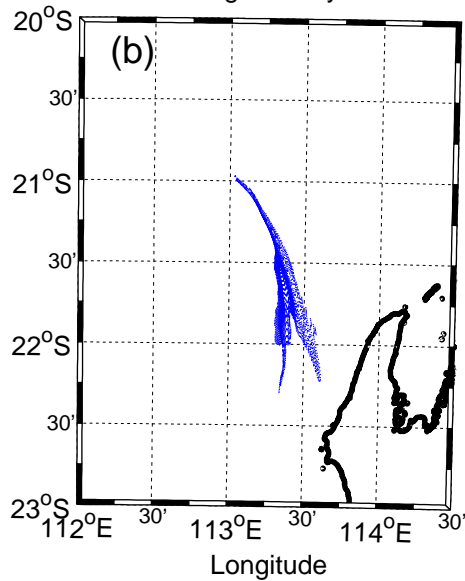

Ningaloo(backward) day0

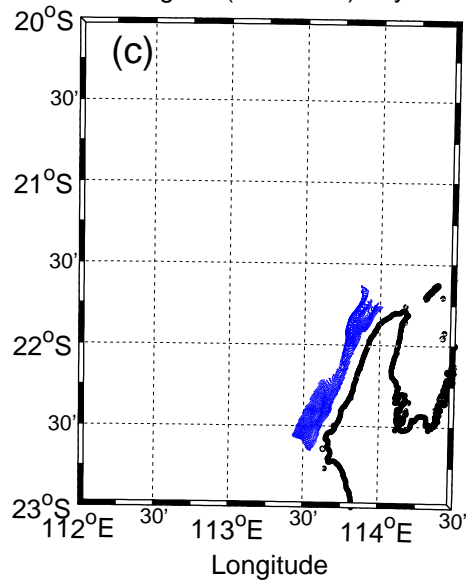

Supplement: S1 Fig — (a) shows the initial positions of all particles at day 0; (b) shows the final positions of all particles at day 5; (c) shows the positions of particles at day 0 after 5 days of backward particle tracking from (b). (PDF) [file pone.0145822.s001.pdf]

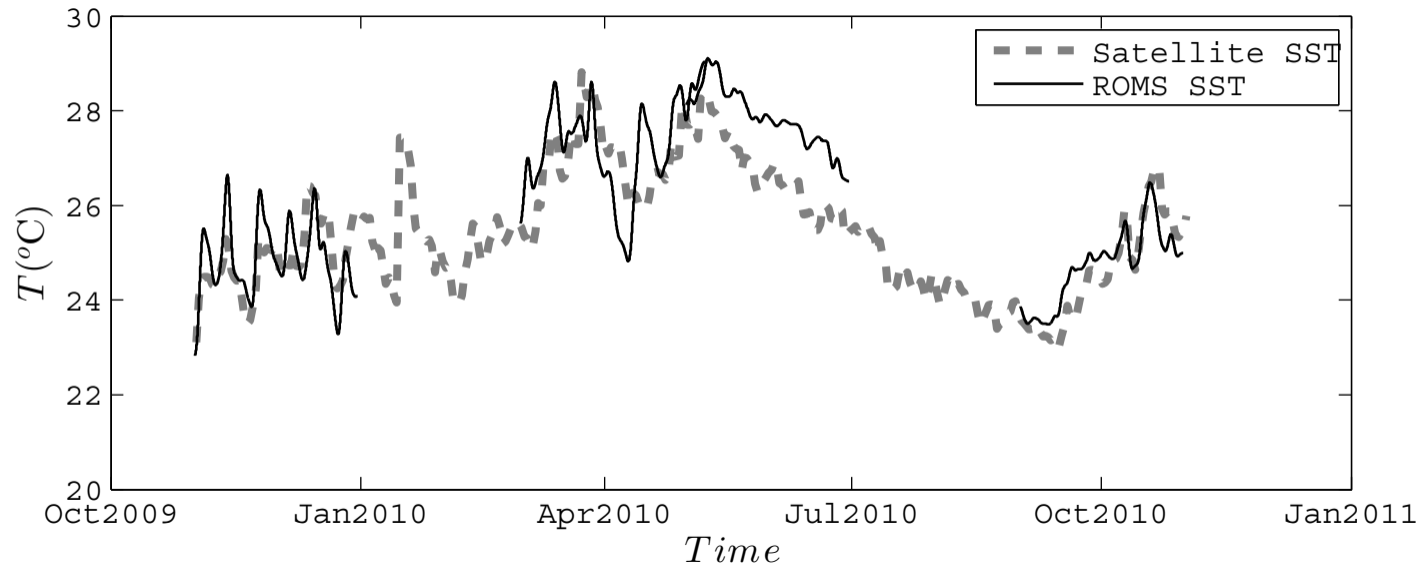

Supplement: S2 Fig — The bold dash line is the satellite SST, and the thin solid line is the modelled SST. (PDF) [file pone.0145822.s002.pdf]

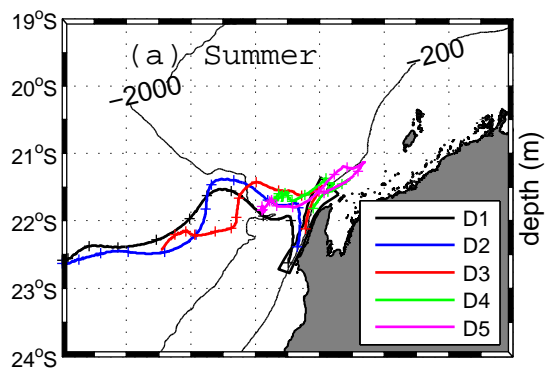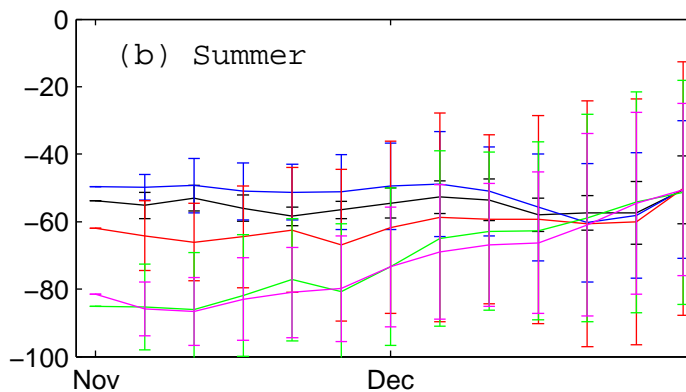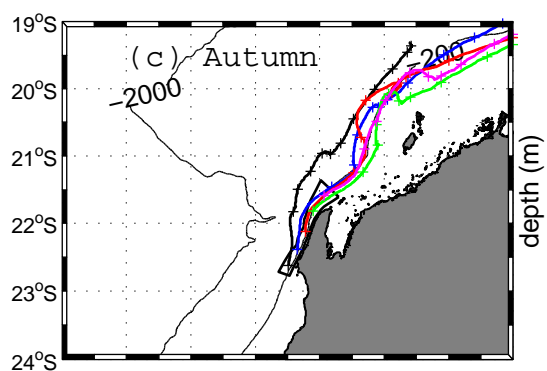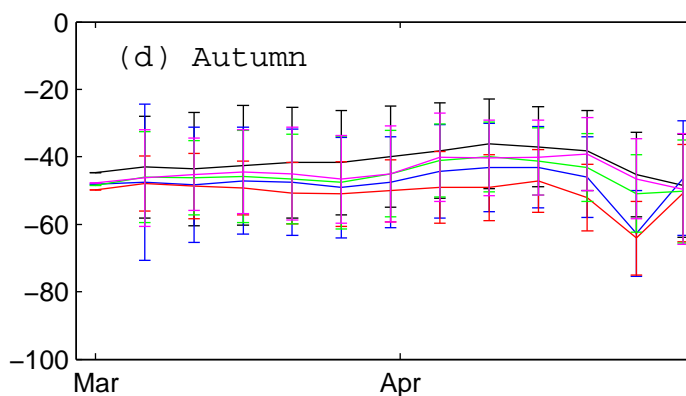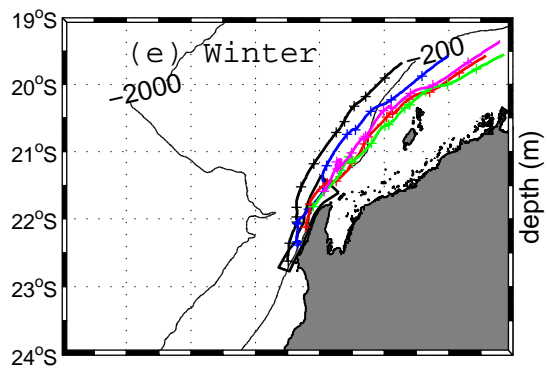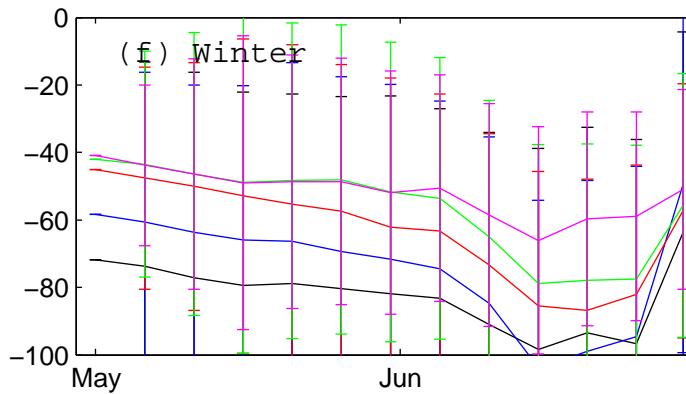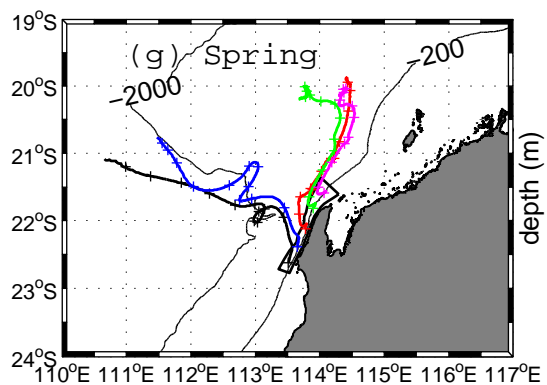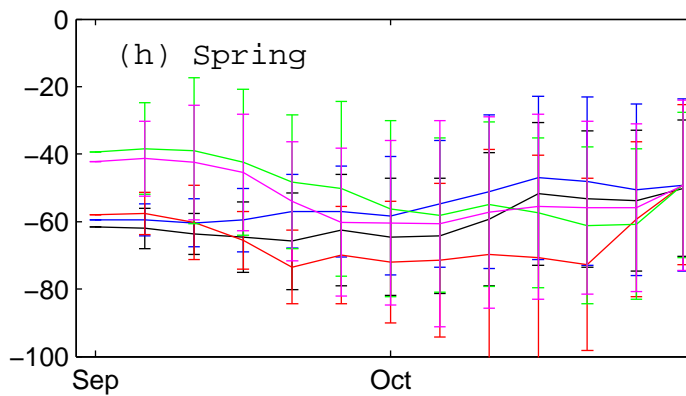

Supplement: S3 Fig — (a, c, e, g) show the mean pathways of particles initialized on the Ningaloo shelf at 50 m depth during summer, autumn, winter and spring, respectively; (b, d, f, h) show time series of the average and standard deviation (error bars) of the depth of the particles every 5 days. (PDF) [file pone.0145822.s003.pdf]
